# Perineural invasion in solid tumors: biological foundations and the emerging integration of machine learning and artificial intelligence

**DOI:** 10.3389/fonc.2026.1801005

**Published:** 2026-04-22

**Authors:** Hisham F. Bahmad, Yasamin Mirzabeigi, Youssef K. Elajami, Wassim Abou-Kheir, Navya Nair

**Affiliations:** 1 Department of Pathology and Laboratory Medicine, University of Miami Miller School of Medicine, Miami, FL, United States; 2 Miami Dade College, Miami, FL, United States; 3 Department of Anatomy, Cell Biology and Physiological Sciences, Faculty of Medicine, American University of Beirut, Beirut, Lebanon; 4 Division of Gynecologic Oncology, Sylvester Comprehensive Cancer Center, Miami, FL, United States; 5 Department of Obstetrics, Gynecology, and Reproductive Sciences, University of Miami Miller School of Medicine, Miami, FL, United States

**Keywords:** artificial intelligence, digital pathology, machine learning, perineural invasion, review, solid tumors

## Abstract

Perineural invasion (PNI) represents a distinct route of cancer spread in many solid tumors. Its presence correlates with aggressive tumor behavior, local recurrence, neuropathic symptoms, and reduced survival across selected tumor types, including pancreatic, prostate, head and neck, colorectal, and gynecologic malignancies, among others. Despite its prognostic value, PNI remains inconsistently detected and reported, and incompletely integrated into the College of American Pathologists (CAP) cancer protocols and clinical decision-making. Over the last decade, advances in tumor-nerve biology have reframed PNI as an active, bidirectional phenomenon driven by molecular crosstalk between cancer cells, Schwann cells, neurons, and the surrounding tumor microenvironment (TME). Parallel advances in digital pathology, machine learning (ML), and artificial intelligence (AI) have opened new opportunities to standardize PNI detection and quantify its extent. This review provides a synopsis of current knowledge on the biological mechanisms and clinical relevance of PNI in solid tumors, with the emerging integration and application of ML- and AI-assisted approaches in histopathology and molecular profiling to advance detection and potential therapeutic targeting of PNI.

## 1 Introduction

Perineural invasion (PNI) describes tumor growth in, around, and along nerves, representing a distinct pathway of cancer spread with significant clinical implications (1, 2). While straightforward when well-formed nerves are clearly wrapped or penetrated by carcinoma, many clinically meaningful cases present diagnostic challenges. Nerves may be small, crushed, tangentially sectioned, or obscured by inflammation and reactive stroma. Sampling limitations can miss critical tumor-nerve interfaces, and even definitions vary across studies and subspecialties, contributing to underreporting, inconsistent documentation, and limited quantification (3–5).

These diagnostic challenges have real clinical consequences. PNI independently predicts aggressive behavior and reduced survival across multiple malignancies (2, 6). In pancreatic ductal adenocarcinoma (PDAC), where PNI prevalence approaches 80-90%, it drives local recurrence, pain, and early treatment failure (7–9). In head and neck squamous cell carcinoma (HNSCC), PNI correlates with locoregional recurrence and influences surgical and radiation planning, particularly when large nerves are involved (10–12). In colorectal cancer (CRC), meta-analyses demonstrate PNI as an adverse prognostic feature with particular relevance in stage II disease, where it informs adjuvant therapy decisions (13–15). In prostate cancer (PCa), the prognostic significance of PNI on biopsy has been debated (16, 17), yet growing evidence supports its association with adverse outcomes in defined clinical contexts (16, 18, 19).

The biological understanding of PNI has fundamentally shifted. PNI reflects active “neurotropism” rather than passive extension, driven by bidirectional signaling between cancer cells and the neural components (1, 20). Tumor cells migrate toward nerves through chemotactic and neurotrophic cues: nerve growth factor-tropomyosin receptor kinase A (NGF-TrkA) axis, glial cell line-derived neurotrophic factor-RET receptor tyrosine kinase (GDNF-RET) pathway, and C-X-C motif chemokine ligand 12 (CXCL12)-C-X-C chemokine receptor 4 (CXCR4) (CXCL12-CXCR4) chemokine axis, while Schwann cells and macrophages actively facilitate invasion through matrix remodeling, immune modulation, and creation of migratory tracks (20–22). These mechanistic insights highlight therapeutic opportunities and underscore the need for better measurement.

## 2 Definitions and reporting challenges

### 2.1 What counts as PNI?

A practical definition used in many studies requires a tumor within any layer of the nerve sheath (endoneurium, perineurium, or epineurium) or a tumor encasing at least 33% of the nerve circumference (23–25). Yet thresholds vary, complicating cross-cohort comparisons and limiting model training (3, 4).

### 2.2 Why is PNI missed or underreported?

Detection of PNI may be affected by sampling (small nerves, limited levels, fragmented specimens), section orientation (tangential cuts that mimic wrapping), confounding factors (perineuritis, reactive fibroblasts, therapeutic effects), and time constraints in routine workflow (3–5, 26). Even when identified, PNI is commonly reported in a binary manner, with many pathologists indicating only its presence or absence. Yet, it has been proven that both the extent and location (intratumoral versus extratumoral, unifocal versus multifocal, large-nerve involvement) provide additional prognostic information (23, 27–29). In prostate biopsies, interobserver agreement for PNI (kappa 0.67-0.75) is substantial but imperfect, with common pitfalls including misidentification of stromal bundles or smooth muscle as nerves and disagreement about tumor-nerve distance (3). In gastric cancer, double immunohistochemical (IHC) staining (labeling both nerves and cancer cells) detected PNI in 65% of cases, compared with 38% with hematoxylin and eosin (H&E) alone; agreement was particularly low in diffuse-type tumors (kappa 0.28) (26).

### 2.3 PNI reporting standards

The College of American Pathologists (CAP) and World Health Organization (WHO) have established comprehensive protocols for reporting PNI across multiple tumor types (including laryngeal and cutaneous squamous cell carcinoma (SCC), PCa, CRC, gallbladder cancer, and PDAC), though their approaches differ in scope and implementation (30–35). Both organizations recognize PNI as a clinically significant feature for risk stratification, but CAP generally designates PNI reporting as optional, though recommended, while WHO more directly incorporates PNI into diagnostic criteria and staging systems (25, 36–42). For instance, the CAP explicitly acknowledges that PNI has been associated with extraprostatic extension (EPE), though its independent prognostic significance remains debated (36). Also, while the CAP CRC protocol specifies PNI as a required element (30), the National Comprehensive Cancer Network (NCCN) guidelines for stage II colon cancer identify PNI as a poor prognostic feature, warranting consideration of adjuvant chemotherapy (13, 14, 43).

## 3 Biological mechanisms of perineural invasion

PNI occurs at the tumor-nerve interface, involving neurons, Schwann cells, immune cells, and the extracellular matrix (ECM). Mechanisms differ by tumor type, but several themes recur.

### 3.1 Neurotrophic NGF/TrkA and GDNF/RET signaling pathways

Neurotrophins promote neural invasion and pain. In PDAC, increased NGF and TrkA expression correlate with PNI and pain, and experimental disruption of NGF signaling reduces neural invasion in models (21, 44, 45). The GDNF-RET axis is particularly exciting, where endoneurial macrophages secrete GDNF, activating RET on pancreatic cancer cells and promoting PNI (21, 22, 44). These pathways are targetable and measurable, making them attractive therapeutic candidates.

### 3.2 Chemotaxis: CXCL12/CXCR4 and CCL2/CCR2 axes

Chemokine gradients act as navigation cues. The CXCL12/CXCR4 pathway promotes pancreatic cancer cell migration toward nerves and is associated with PNI in human samples; pathway inhibition reduced nerve invasion in preclinical work (6, 44). CCR2-expressing inflammatory monocytes are preferentially recruited to sites of PNI, where they differentiate into macrophages and potentiate nerve invasion through cathepsin B-mediated processes (22). CCL2 released from Schwann cells drives this recruitment, and interruption of the CCL2-CCR2 signaling significantly impairs PNI (22, 46, 47).

### 3.3 Schwann cells: active guides rather than passive barriers

Schwann cells have emerged as central players in PNI. Upon interacting with cancer cells, they induce the formation of cancer cell protrusions and intercalate between tumor cells, promoting contact-dependent invasion mediated by neural cell adhesion molecule 1 (NCAM1) (20, 48, 49). Schwann cells undergo phenotypic reprogramming to a reparative state, secreting neurotrophins, chemokines (including CCL2 and fibroblast growth factor 17 (FGF17)), and ECM-degrading enzymes (such as cathepsin S and matrix metalloproteinase-12 (MMP-12)) that facilitate cancer cell migration (20, 50–53). Interestingly, single-cell transcriptomics have identified distinct Schwann cell subsets, including transforming growth factor beta-induced protein (TGFBI)+ Schwann cells that locate at the leading edge of neural invasion and correlate with poor survival (54).

### 3.4 Axon guidance molecules and matrix remodeling: Sema4D/Plexin-B1

Axon guidance pathways classically studied in development are repurposed by tumors. Semaphorin 4D (Sema4D) and its receptor, Plexin-B1, are crucial components of the axon guidance pathways that are frequently repurposed by neurotropic cancers to promote PNI (6). In cancer, these pathways are repurposed to create bidirectional communication between cancer cells and nerves. Cancer cells expressing Sema4D interact with Plexin-B1 receptors on nerves (and vice versa), promoting cancer cell migration toward nerves and enhancing invasiveness through activation of downstream signaling pathways, including PI3K/Akt and MAPK/ERK (55–57).

Once cancer cells are attracted to nerves via axon guidance signals, they can physically penetrate the perineural barriers, wherein MMPs and other proteolytic enzymes become critical (58–60). Cancer cells, Schwann cells, and stromal fibroblasts upregulate MMPs (particularly MMP-1, MMP-2, and MMP-9) and cathepsins that degrade the ECM components surrounding nerves (22, 52, 61). Perineural fibroblasts specifically express MMP-2 at sites of PNI but not at uninvolved nerves (58). Additionally, TGF-β1 secreted by cancer cells induces epithelial-to-mesenchymal transition (EMT) in perineural epithelial cells, causing them to upregulate MMP-3 and MMP-9, thereby degrading their own protective barrier (61).

The MMP1/protease-activated receptor-1 (PAR1)/substance P (SP)/neurokinin 1 receptor (NK1R) paracrine loop exemplifies how these processes integrate, where MMP-1 from cancer cells activates receptors on nerves, triggering substance P release, which then promotes cancer cell migration and invasion (59). Similarly, axon guidance molecules like SEMA3D promote PNI, while cancer cells simultaneously employ ECM-degrading enzymes to physically navigate through the perineural space (56). In essence, axon guidance pathways inform and guide the cancer cells on “where to go” along the nerves, while matrix remodeling enzymes enable them to “break through” the physical barriers to get there, creating the permissive “paths” along nerve sheaths (20, 54, 62).

## 4 Clinical impact across solid tumors

### 4.1 Pancreatic ductal adenocarcinoma

PNI is extremely common in PDAC and contributes to pain and local recurrence (7–9). In margin-negative and lymph node-negative (R0/N0)-resected PDAC (traditionally considered favorable), PNI was shown to be the strongest predictor of overall survival (OS), with a hazard ratio (HR) of 2.24 (95% CI: 1.52-3.30; *P* < 0.001) (7). Severe PNI (infiltration of nerves ≥3mm, massive invasion, or nerve necrosis) independently predicts both recurrence (HR 2.08; *P=*0.006) and survival (HR 3.304; *P* = 0.014), with these patients deriving the greatest benefit from adjuvant therapy (7, 63). That being said, neural invasion severity is a powerful independent factor influencing OS and local recurrence, with higher severity scores leading to significantly more and earlier local recurrence than distant metastasis (64). While these prognostic associations are among the most strongly established in PDAC, the extent to which neural invasion severity scoring systems are applicable to other solid tumors remains under investigation, with emerging evidence supporting similar prognostic stratification in head and neck and colorectal cancers.

### 4.2 Head and neck cancer squamous cell carcinoma

PNI is a well-established adverse factor in HNSCC. A meta-analysis including 18 studies with 3, 894 patients found that PNI was significantly associated with worse OS (HR 2.8, 95% CI: 1.88-4.16), disease-free survival (DFS) (HR 2.42, 95% CI: 1.92-3.05), and disease-specific survival (DSS) (HR 2.60, 95% CI: 1.86-3.63) (11). Recent studies continue to support PNI as a meaningful prognostic marker and emphasize the need to integrate PNI status into risk stratification (10, 11, 65). Beyond microscopic PNI, perineural spread along named cranial nerves changes operative and radiation fields (12, 27).

### 4.3 Colorectal cancer

PNI is a well-established adverse prognostic factor in stage II colon cancer. In multivariate analysis, PNI has been shown to be an independent prognostic factor for OS, DFS, and DSS. For stage II colon cancer patients, those with PNI have significantly worse 5-year DFS compared to those without PNI (29% vs. 82%; *P* = 0.0005) (43, 66–68). This is especially relevant in stage II CRC, where PNI can shift a patient into a higher-risk category considered for adjuvant therapy (13–15).

The anatomical location of PNI, whether it occurs within the bowel wall (intramural) or beyond it (extramural), also represents a critical refinement in the prognostic stratification for stage II colon cancer. In a landmark study of 1, 130 stage II colon cancer patients, extramural invasion (versus intramural invasion) was associated with significantly worse prognosis, with multivariate analysis confirming extramural invasion as a significant independent prognostic factor for both DFS and OS (29). Importantly, intramural invasion showed no significant difference in outcomes compared with patients without invasion, suggesting that pathologic reports on the location of lymphatic, vascular, and PNI might be helpful for predicting prognosis and determining the need for adjuvant chemotherapy in stage II colon cancers.

Similarly, PNI depth has prognostic implications. Deep PNI (defined as PNI in the subserous plexus) was associated with worse OS (HR 3.546, 95% CI: 2.307-5.449; *P* < 0.001) and DFS (HR 2.921, 95% CI: 2.032-4.198; *P* < 0.001) compared to non-PNI, whereas superficial PNI (in the submucosal and myenteric plexus) showed no significant difference from non-PNI (28).

### 4.4 Prostate cancer

The prognostic meaning of PNI on prostate biopsy has been argued for decades. A systematic review published back in 2007 concluded that the weight of evidence supports the prognostic significance in defined groups, though the study heterogeneity limits precise risk estimates (19). In fact, quantification of PNI (such as multifocal PNI or higher PNI density per tumor length) may better capture risk than a simple present/absent call.

The anatomical location of PNI within the prostate biopsy core also represents an important spatial refinement in prognostic assessment for PCa (18, 69, 70). PNI detected within 1 mm of the biopsy core tip independently predicted worse postoperative prognosis (HR 3.228; *P* = 0.002) in a study of 180 patients with a single focus of PNI (70). Remarkably, there were no significant differences in clinicopathologic features between the <1 mm versus ≥1 mm groups, including total tumor length on biopsy, estimated tumor volume on prostatectomy, tumor grade, pathologic T or N category, or surgical margin status, suggesting that the spatial location of PNI provides independent prognostic information beyond traditional pathologic parameters (70).

This finding builds upon other quantitative assessments of PNI. Multifocal PNI (multiple foci per biopsy) was associated with significantly worse outcomes compared to single-focus PNI, with multifocal PNI showing an HR of 5.48 (*P* < 0.001) for biochemical recurrence (18). Similarly, >1 PNI per 10 mm of tumor was an independent predictor of recurrence (HR 3.96; *P* < 0.001). PNI involving multiple sextant sites on biopsy was also independently associated with disease progression (HR 1.556; *P* = 0.03) and correlated with higher-grade disease and larger tumor volumes at radical prostatectomy (18).

The biological rationale for location-based prognostic significance likely relates to the proximity to the prostatic capsule and extraprostatic neural plexuses. PNI near the biopsy core tip may indicate cancer cells approaching or at the prostatic margin, where they can access larger caliber nerves and potentially breach the capsule. Indeed, a study demonstrated that PNI epithelial cells within 1 mm of the prostate edge exhibited an activated metastatic phenotype with increased expression of amoeboid signaling molecules (EphA2, pEphA2^s897^, pMLC2) and mitochondrial defects (loss of Complex I and IV and gain of mitochondrial mass (TOMM20)), consistent with a migrational switch (71).

### 4.5 Gynecologic oncology

PNI is an emerging prognostic factor across multiple gynecologic malignancies, with the strongest evidence in cervical and vulvar cancers. The prevalence and clinical significance vary by tumor type, but PNI consistently correlates with aggressive tumor biology and worse survival outcomes.

#### 4.5.1 Cervical cancer

PNI is detected in 7.0-35.1% of cervical carcinomas and represents a significant independent prognostic factor (72–74). A meta-analysis of seven studies including 1, 561 women demonstrated that PNI was associated with significantly worse 5-year OS (risk ratio 2.79, 95% CI: 1.67-4.66) and 5-year DFS (risk ratio 2.16, 95% CI: 1.30-3.59) (74). After multivariate adjustment, PNI remained an independent predictor with an HR of 3.25 (95% CI: 1.09-9.74) for OS and 2.50 (95% CI: 0.66-9.46) for DFS (74). PNI correlates with other high-risk features, including deep stromal invasion, lymphovascular space invasion (LVSI), larger tumor size, lymph node metastasis, parametrial invasion, and positive resection margins (72–74).

The anatomical location of PNI provides additional prognostic implications. Parametrial PNI is particularly significant; in early-stage cervical cancer patients with negative lymph nodes, parametrial PNI independently predicted poor outcome and more than doubled the risk of recurrence in patients with tumors >4 cm (75). A multicenter study of 1, 208 cases from China found that PNI was an independent risk factor for both 5-year OS and DFS, with the study notably revealing a false negative rate of 33.33% for PNI diagnosis, suggesting that PNI may be underreported (73).

PNI has been proposed as an intermediate-risk factor for recurrence, with PNI-positive patients showing similar survival to those meeting Seldis criteria for adjuvant therapy (76). The presence of PNI has also become a contraindication for nerve-sparing radical hysterectomy due to concerns about inadequate tumor resection (77).

#### 4.5.2 Vulvar cancer

PNI is found in 7.6-52.4% of vulvar SCCs and represents one of the most powerful independent prognostic factors in this disease (72, 78). A systematic review and meta-analysis study demonstrated that PNI was significantly associated with decreased OS (HR 2.687; *P* < 0.001), DSS (HR 2.375; *P* = 0.014), and progression-free survival (PFS) (HR 1.757; *P* = 0.001) (78). PNI also strongly correlates with depth of invasion, LVSI, tumor size, advanced stage, and nodal involvement (72, 78). In a large single-institution study of 421 patients, those with PNI had significantly shorter median OS (25.5 vs. 94.3 months; *P* < 0.001) and PFS (17.5 vs. 29.0 months; *P* = 0.004), with PNI conferring a greater than 2.5-fold increased risk of death even after adjusting for stage (79).

#### 4.5.3 Ovarian cancer

Limited data exist on PNI in ovarian cancer, but emerging evidence suggests prognostic significance. In high-grade serous ovarian cancer, patients with PNI-positive tumors had significantly shorter OS compared to PNI-negative patients (80).

#### 4.5.4 Endometrial cancer

While LVSI is well-established as a prognostic factor in endometrial cancer and incorporated into risk stratification models, PNI has received limited attention in this disease (81). Preclinical studies have demonstrated that dorsal root ganglion neurons promote PNI of endometrial cancer cells via the glutamate receptor GluR2, suggesting a potential mechanism for nerve-tumor interaction (82). However, PNI is not currently included in standard pathology reporting or risk stratification for endometrial cancer, and further clinical studies are needed to establish its prognostic significance.

### 4.6 Biliary tract cancers

PNI is common across biliary tract malignancies. Prevalence varies by the site where perihilar cholangiocarcinoma (pCCA) has the highest rates, followed by distal cholangiocarcinoma (dCCA), gallbladder cancer (GBC), and intrahepatic cholangiocarcinoma (ICC) (83–87). The anatomical proximity of the biliary structures to the hepatoduodenal ligament and celiac nerve plexuses creates a suitable microenvironment for nerve-tumor interactions. In a multicenter study including 1, 095 resected ICC patients, PNI independently predicted reduced DFS (HR 1.56; *P* = 0.019) and OS (HR 1.74; *P* = 0.007), even in early-stage (T1-2) and node-negative disease (87). Similarly, in pCCA, an international multicenter study of 435 patients found PNI independently associated with worse OS (HR 1.52), with adjuvant chemotherapy providing a significant survival benefit specifically among node-negative patients with PNI (50.8 months vs. 28.6 months; *P* = 0.044) (88). In GBC, PNI has been shown to be strongly associated with extrahepatic bile duct invasion, raising the question of whether bile duct resection should be recommended in such cases, though evidence suggests no significant survival advantage from this approach (89).

Subcellular spatial transcriptomics of dCCA has revealed enrichment of Schwann cells, M2-like macrophages, and cancer-associated fibroblasts in PNI-high tumors, with the LAMB3-DAG1 axis identified as a potential mediator of tumor cell-Schwann cell interaction (62). In GBC, tumor-derived extracellular vesicles containing O-GlcNAcase trigger neuronal necroptosis via the RIPK1/RIPK3/MLKL axis, with subsequent HMGB1 (High Mobility Group Box 1) release engaging RAGE (Receptor for Advanced Glycation End) on tumor cells to establish a self-amplifying PNI loop (90).

### 4.7 Gastric cancers

PNI is detected in approximately 40% of gastric cancers and serves as an independent prognostic factor (91). A meta-analysis of 30, 590 patients reported a pooled adjusted HR of 1.484 for OS, while a second meta-analysis of 7, 004 patients confirmed worse survival (HR 1.69) and significant associations with deeper invasion, lymphatic invasion, vascular invasion, and lymph node metastasis (91, 92). Interestingly, PNI has also emerged as a potential upstaging factor in node-negative (N0) gastric cancer, where N0 patients with PNI had survival curves similar to N1 patients (93). After neoadjuvant chemotherapy, residual PNI on surgical pathology independently predicted survival (HR 2.11) (94), with concurrent lymphovascular invasion and PNI conferring even greater risk (HR 2.62 for OS) (95).

## 5 AI and machine learning for PNI detection and quantification

Standard, human-based histopathologic examination and assessment have limited sensitivity for detecting PNI, with careful re-review of H&E-stained slides shown to increase detection from 30% to 62%, and IHC staining with S100 further improving detection to 82% in oral cavity SCC (OCSCC) (96, 97). Also, interobserver variability remains substantial, with kappa values of 0.67-0.75 among expert pathologists, and PNI is often predominantly reported as a binary variable despite evidence that quantitative features (including nerve caliber, number of involved nerves, tumor-nerve distance, and spatial distribution, among others) significantly influence prognosis (3, 23, 98). The lack of standardized reporting criteria, suboptimal tissue sampling, and the time-intensive nature of comprehensive PNI assessment further limit its integration into clinical decision-making (99). Artificial intelligence (AI) and machine learning (ML) address these challenges by enabling consistent, high-throughput screening of large tissue areas, reducing diagnostic variability through application of uniform criteria, and extracting quantitative morphologic features that potentially can correlate with outcomes and molecular profiles (1, 98, 100, 101). Deep learning algorithms have demonstrated performance comparable to or exceeding pathologist concordance, with diagnostic accuracy (area under the curve; AUC) of 98% for PNI detection in prostate biopsies and the ability to identify occult PNI missed on initial review, while simultaneously reducing analysis time by 15-24% and enabling integration of spatial, molecular, and clinical data for precision oncology applications (97, 98, 100, 101).

### 5.1 Interobserver variability: the human baseline

The reproducibility of PNI assessment among pathologists is moderate at best, establishing the benchmark against which AI systems are evaluated. In prostate needle biopsies, interobserver agreement among four experienced urological pathologists yielded kappa values of 0.67-0.75 (mean 0.73), with complete agreement in only 34.0% of cases and disagreement in 24.5% (3). Common diagnostic pitfalls included misidentifying stromal bundles or smooth muscle as nerves, uncertainty about tumor-nerve proximity thresholds, and rare confusion with collagenous micronodules (3). For comparison, AI concordance with pathologists (kappa 0.74) matched inter-pathologist concordance (kappa 0.68-0.75) in the same tissue type (98). In CRC, PNI assessment showed only 26% discordance (kappa 0.530) for LVSI, though this improved for other features (102). The variability extends beyond detection to quantification, where even experienced gastrointestinal pathologists showed only poor to moderate agreement (kappa 0.29-0.59) for extramural venous invasion, a closely related histologic feature (103).

### 5.2 Deep learning architectures for PNI detection

Current AI approaches employ convolutional neural networks (CNNs) as the foundational architecture for PNI detection, with variations in implementation strategy (101, 104, 105). The most successful models use two-stage semantic segmentation that mimics pathologist decision-making: first identifying nerve structures, then detecting tumor-nerve interactions (100, 101). The Domain-KEY algorithm for OCSCC exemplifies this approach, achieving 89.01% diagnostic accuracy compared to 61.94% for single-stage ResNet-based classifiers, with a mean accuracy of 97.50% on whole-slide images (WSIs) and 96% accuracy when validated on oropharyngeal and hypopharyngeal cancers (101). For pancreatic cancer, a similar two-stage approach using manually segmented training data from only six cases achieved 88% sensitivity and 78% specificity for nerve detection, with tumor detection at 54% sensitivity and 85% specificity (100). Fully convolutional networks (FCNs) have emerged as particularly effective for pathology image segmentation due to their computational efficiency and generalizability (105). More recent architectures incorporate ensemble models combining DenseNet-121, Inception-ResNet-V2, and DeepLabV3Plus, trained end-to-end for each task (106). Domain adaptation using expectation-maximization algorithms enables models trained on one cancer type to generalize across others: a model trained on PCa achieved Dice coefficients of 0.82 for PCa and 0.79 for head and neck cancer after domain adaptation (107). Graph convolutional networks represent an emerging approach that captures spatial relationships between histological entities, encoding tissue topology and inter-entity interactions beyond what patch-based CNNs can achieve (108).

### 5.3 Quantitative features and spatial metrics

AI enables the extraction of quantitative metrics that transcend binary PNI reporting. The most clinically validated spatial metric is tumor-nerve distance, where incorporating proximity thresholds increased PNI detection from 52% to 81% of PDAC cases, with pathologist review requiring only an average of 24 seconds per case (100). Beyond simple distance, AI models can quantify nerve caliber (diameter measurements), number of involved nerves per section (focal: 1 nerve, moderate: 2–4 nerves, extensive: ≥5 nerves), anatomic location relative to tumor (intratumoral, advancing edge, extratumoral), and depth of invasion (23). These quantitative assessments address long-standing clinical questions related to prognosis. Indeed, the prognostic relevance of specific quantitative features may vary across tumor types; for instance, extensive PNI proved to be more prognostically significant than traditional large-caliber PNI (≥0.1mm diameter) in cutaneous SCC (23), whereas nerve caliber may carry greater weight in PDAC. Further cross-tumor validation of these metrics is needed to establish which quantitative parameters are universally applicable versus cancer type-specific.

Pathomics approaches extract hundreds of morphological features from H&E-stained slides using automated pipelines like CellProfiler (109). In a study on CRC, 615 histopathological features were extracted, with 10 selected via LASSO (Least Absolute Shrinkage and Selection Operator) regression to build diagnostic models achieving AUCs of 0.996 (training), 0.935 (testing), and 0.918 (external validation) using the LightGBM algorithm (109). These features capture cellular architecture, nuclear morphology, spatial organization, and textural patterns invisible to human observers (110, 111). Habitat-based analysis using K-means clustering divides tumors into subregions with distinct imaging characteristics, with models analyzing intratumoral, peritumoral (3mm beyond tumor), and subregion features achieving AUCs of 0.912 (training) and 0.880 (external validation) for rectal cancer PNI prediction (112).

A methodological consideration relevant to these pathomic and radiomic studies is the widespread use of LASSO regression for feature selection. While LASSO effectively reduces high-dimensional feature spaces, it has well-known limitations when features are highly correlated. This is a common scenario in radiomics and pathomics, where many extracted features capture overlapping spatial, textural, or morphological information. In the presence of multicollinearity, LASSO tends to subjectively select one feature from a group of correlated variables while discarding the others, leading to feature selection bias. That being said, different random seeds or data splits can yield substantially different feature subsets without meaningful differences in predictive performance. This complicates biological interpretation, as the selected features may not represent the most biologically relevant variables. Elastic net regularization, which combines L1 and L2 penalties, offers a partial solution by tending to retain groups of correlated features. Importantly, the resampling and cross-validation strategies used during feature selection are also critical but underreported. Performing feature selection on the full dataset before cross-validation (a form of data leakage) can inflate performance estimates; proper nested cross-validation, in which feature selection is repeated within each fold, provides more realistic estimates but is computationally demanding and infrequently implemented.

### 5.4 Performance across cancer types

AI models show high, often expert-level performance across diverse malignancies, though metrics vary by tissue type and clinical context. For PCa, deep neural networks achieved an AUC of 0.98 (95% CI: 0.97-0.99) for PNI detection in needle biopsies, with sensitivity 0.87, specificity 0.97, and negative predictive value 0.99 (98). Combined deep learning-radiomics-clinical models integrating biparametric MRI reached AUCs of 0.914 (training) and 0.848 (validation) (113). In PDAC, radiomics models for PNI prediction achieved AUCs of 0.899 (training) and 0.813 (validation), improving to 0.945 and 0.881 when integrated with clinical features including tumor diameter, CA19-9, glucose, and lymph node status (114). Fully automated CT-based models for extrapancreatic PNI reached AUCs of 0.87 (training) and 0.83 (external validation), with significant prognostic stratification (115).

For head and neck cancer, a 44-gene expression signature trained via random forest achieved high accuracy for PNI prediction, including occult invasion not detected on initial pathology review (116). Gastric cancer radiomics models combining LASSO feature selection with multiple ML algorithms achieved AUCs of 0.89 (training), 0.84 (internal testing), and 0.82 (external testing) (117). On the other hand, CRC pathomics signatures using LightGBM demonstrated exceptional performance with AUCs exceeding 0.91 across validation cohorts (109).

An important caveat when interpreting these performance metrics is the absence of a standardized evaluation framework across the cited studies. The reported models differ substantially in their data sources (histopathology versus radiology versus gene expression), study designs (retrospective single-center versus multicenter), sample sizes, outcome definitions, and validation strategies, making direct cross-study comparisons of AUC values potentially misleading. For instance, a histopathology-based model detecting PNI on tissue sections addresses a fundamentally different task than a radiomics model predicting PNI status from preoperative imaging, even though both may report similar AUCs. The CAP has published recommendations for performance evaluation of ML in pathology, emphasizing the need for standardized reporting of study population characteristics, reference standards, model development processes, and performance metrics beyond discrimination (118). Adoption of structured reporting guidelines, such as TRIPOD-AI (Transparent Reporting of a multivariable prediction model for Individual Prognosis Or Diagnosis for AI) or the CAP recommendations, would greatly enhance cross-study interpretability and should be a priority for future PNI-related AI research (119).

### 5.5 Integration of morphologic, molecular, and clinical data

The most powerful AI applications integrate multiple data modalities to improve prediction and reveal biological mechanisms. Transcriptomic classifiers identify PNI-associated gene expression programs enriched for neural-like, pro-survival, and invasive signatures, with many differentially expressed genes mapping specifically to malignant cells rather than nerves (97). Radiomics-clinical integration consistently outperforms either modality alone. In PCa, for example, deep learning-radiomics-clinical (DLRC) models integrating MRI-based habitat analysis with clinical factors reached AUCs of 0.999 (training), with sensitivity 1.0 and specificity 0.955 (120).

SHAP (SHapley Additive exPlanations) is a widely adopted explainable AI technique that quantifies each feature’s contribution to ML model predictions, thereby enhancing interpretability and supporting clinical decision-making (118, 121–123). SHAP analysis has identified PNI as an important predictive feature in several clinical machine learning applications. In this context, SHAP serves two distinct roles. First, it identifies which features most strongly predict the presence of PNI. For example, in intrahepatic cholangiocarcinoma, SHAP analysis revealed that tumor number, tumor size, and lymph node metastasis were the top three predictive features (124). In gallbladder carcinoma, SHAP ranked total bilirubin, CA19-9, imaging-detected liver invasion, vascular invasion, and TNM staging as the most important PNI predictors (125, 126). ML models with SHAP interpretation enable preoperative PNI risk stratification, potentially guiding decisions about neoadjuvant therapy, surgical extent, and adjuvant treatment (114). Notably, SHAP’s model-agnostic nature allows it to explain predictions from diverse algorithms, including XGBoost, LightGBM, support vector machines, and neural networks—all of which have been successfully applied to PNI prediction (114, 118, 124, 125).

However, important methodological qualifications should accompany the interpretation of SHAP-based explanations. SHAP values are model-specific where features ranked as important by one algorithm (e.g., XGBoost) may not retain the same ranking in another (e.g., random forest or neural network) trained on the same data. In small-dataset settings, which characterize many PNI prediction studies, SHAP values can be unstable across reruns or cross-validation folds, with feature rankings shifting substantially depending on data partitioning. Additionally, SHAP quantifies each feature’s contribution to prediction within a correlational framework; it does not establish causal relationships. A feature identified as highly predictive of PNI (e.g., tumor size or lymph node status) may be a confounding correlate rather than a mechanistic driver. Interpreting SHAP outputs as causal explanations can mislead clinical reasoning. Future studies should assess SHAP stability through repeated resampling and consider complementary interpretability methods (e.g., permutation importance, LIME, or attention mechanisms in deep learning) to triangulate feature importance findings.

### 5.6 Workflow integration and clinical utility

AI tools may function as decision support systems that highlight regions of interest (ROIs) for pathologists to review, rather than autonomous diagnostic platforms (98, 100, 101). Researchers from Taiwan developed a deep learning-based human-enhanced tool, termed the domain knowledge enhanced yield (Domain-KEY) algorithm, for identifying PNI in digital slides. The Domain-KEY algorithm successfully reduced diagnostic time by 15.0% to 23.7% for experienced pathologists while maintaining 97.5% accuracy (101). For prostate biopsies, AI reduced the number requiring detailed PNI assessment by flagging high-probability cases, with NPV 0.99 enabling confident exclusion (98).

It is important to note that standardization and reproducibility represent the key advantages of ML and AI over manual assessment. AI applies consistent criteria across cases and institutions, addressing the disagreement among expert pathologists (127). Models trained on standardized annotations can be deployed across centers, though domain adaptation may be required for optimal performance in new tissue types or staining protocols (107). The integration of AI-quantified PNI metrics into structured pathology reports could enable PNI to function as a companion diagnostic for therapies targeting tumor-nerve interactions, analogous to PD-L1 for immunotherapy (99).

Nevertheless, limitations and potential side effects of AI-driven PNI detection tools exist, extending beyond technical performance metrics to encompass data quality issues, algorithmic vulnerabilities, workflow integration challenges, and ethical concerns that could impact patient safety and diagnostic accuracy.

### 5.7 Data-related limitations

Data bias and lack of generalizability represent fundamental challenges. AI models trained on datasets from specific institutions, patient populations, or scanning equipment may fail when deployed in different clinical contexts—a phenomenon referred to as “distributional shift” (127). Hidden stratification can occur when training data inadvertently contains confounding variables (e.g., specific staining protocols, scanner types, or demographic characteristics) that the algorithm learns to rely upon rather than true pathological features (127). For PNI detection specifically, the rarity of positive cases creates severe class imbalance. For instance, only 485 of approximately 80, 000 prostate biopsy cores showed PNI in one large study, potentially causing models to underperform on the minority class despite high overall accuracy (98). Additionally, the validation crisis in AI pathology is particularly concerning. Foundation models demonstrate poor generalizability in zero-shot testing scenarios when applied to new datasets without retraining (128). This means algorithms validated at one institution may perform substantially worse when deployed elsewhere, yet many published studies lack rigorous external validation (128, 129).

The implications of class imbalance extend beyond simple underperformance. When PNI-positive cases constitute a small fraction of the training set, models may develop a bias toward the majority class, achieving high overall accuracy while failing to detect the very cases of clinical interest. Techniques to mitigate class imbalance, including oversampling (e.g., SMOTE), undersampling, cost-sensitive learning, and data augmentation, are well-established in the ML literature but are inconsistently reported in the PNI studies reviewed here. The choice of mitigation strategy can substantially influence both model performance and the types of errors produced, yet many studies do not specify whether or how they addressed this issue. Furthermore, the absence of confidence intervals around reported performance metrics limits the ability to assess statistical precision and make meaningful comparisons between models. A model reporting an AUC of 0.89 without confidence intervals cannot be meaningfully distinguished from one reporting 0.91, yet such comparisons are implicitly made throughout the literature. Formal statistical comparisons (e.g., DeLong tests for AUC differences, bootstrapped confidence intervals) are largely absent from the current PNI prediction literature, making it difficult to determine whether apparent differences in performance reflect true model superiority or sampling variability.

Beyond generalizability, several additional methodological concerns warrant consideration when interpreting the AI models reviewed here. First, the reported studies predominantly rely on discrimination metrics (AUC, sensitivity, specificity) while largely omitting calibration assessment, i.e., the degree to which predicted probabilities match observed event rates. A model may achieve a high AUC yet produce poorly calibrated probability estimates, limiting its clinical utility for individual patient risk stratification. Calibration plots, Hosmer-Lemeshow tests, or Brier scores are rarely reported in the PNI prediction literature but are essential for evaluating whether model outputs can meaningfully inform clinical decisions.

Moreover, the validation strategies employed vary considerably across studies. Many models report only internal validation (e.g., random train-test splits from a single institution), which tends to overestimate real-world performance. External validation on independent cohorts from different institutions, scanners, and patient populations provides a more realistic assessment of generalizability but remains uncommon. Prospective validation is largely absent from the current literature. The substantial gaps between training and validation AUCs observed in several of the cited studies (e.g., training AUC 0.996 vs. external validation AUC 0.918 (109); training AUC 0.999 vs. unspecified external validation (118)) raise concerns about overfitting, where models may learn institution-specific or dataset-specific patterns rather than biologically meaningful features. These gaps should be interpreted with caution rather than treated as evidence of strong model performance.

Lastly, the clinical utility remains largely undemonstrated. Decision curve analysis, net reclassification improvement, and randomized impact studies are needed to establish that AI-assisted PNI detection translates into meaningful clinical benefit beyond what existing pathologic assessment provides.

### 5.8 Algorithmic errors and failure modes

We must admit that AI systems can potentially make qualitatively different errors than human pathologists. Common pitfalls in PNI detection include misidentifying stromal bundles or smooth muscle as nerves, and confusion with collagenous micronodules (98). Unlike human errors that often involve oversight or fatigue, AI errors stem from pattern recognition failures that may be systematic and reproducible across similar cases (127, 129). Such unsafe failure modes occur when algorithms fail in unpredictable or dangerous ways (127). For example, an AI tool might confidently misclassify a PNI-positive case as negative (a high-certainty false negative), providing no indication to the pathologist that a closer review is warranted. That being said, the “black-box” nature of deep learning models makes it difficult to understand why specific errors occur, limiting pathologists’ ability to recognize when the algorithm is likely wrong (128, 130).

Moreover, hallucinations in generative AI tools represent an emerging concern, where models may fabricate plausible-sounding but incorrect diagnostic information (128). While less relevant for current PNI detection algorithms, this becomes critical as AI “copilots” like PathChat and SmartPath emerge to provide conversational diagnostic support (128, 131).

### 5.9 Automation bias and deskilling

Automation bias, defined as the tendency to over-rely on automated systems and discount contradictory information, poses a significant further risk (127, 132). Pathologists may defer to AI predictions even when their own assessment suggests otherwise, particularly when workload is high or the AI presents results with apparent confidence. This could lead to misdiagnoses when the algorithm fails. Conversely, widespread AI adoption may instigate pathologist deskilling and dethrilling (132). If AI handles routine PNI screening, pathologists may lose proficiency in detecting subtle cases, reducing their ability to identify AI errors. The removal of intellectually engaging diagnostic work can contribute to “professional burnout” by diminishing pathologists’ sense of meaning and personal accomplishment and increasing their cognitive burden from administrative tasks. This shift fundamentally alters the core professional identity of physicians, who typically enter medicine to solve complex clinical problems and help patients (132).

This combination of reduced intellectual engagement, loss of diagnostic autonomy, and erosion of clinical skills directly impacts the core dimensions of burnout (emotional exhaustion, depersonalization, and reduced personal accomplishment) while undermining the professional identity that provides resilience against burnout (133–135).

### 5.10 Reinforcement of outdated practices

AI models trained on historical data may perpetuate outdated diagnostic criteria or biases present in their training sets (127). For PNI, where reporting criteria lack standardization and understanding of biological significance continues to evolve, algorithms might reinforce inconsistent or obsolete practices rather than incorporating emerging knowledge (99). This creates a paradox in which AI promises standardization but may also standardize incorrect approaches.

### 5.11 Infrastructure challenges

Implementing AI in pathology requires substantial infrastructure investments, including fully digital imaging platforms that can cost hundreds of thousands to millions of dollars, massive data storage capacity to accommodate WSIs that typically range from 1–5 GB each, comprehensive overhaul of legacy IT systems to enable integration with laboratory information systems (LISs), significant workflow modifications, and development of sustainable reimbursement models, all of which create formidable financial and operational barriers to adoption. The transition to digital pathology necessitates high-resolution WSI scanners, which serve as the enabling platform for AI applications but represent a major capital expenditure (136, 137). Storage requirements are particularly challenging. WSIs commonly reach resolutions of 100, 000 × 100, 000 pixels with color information at multiple magnifications and z-stack levels, generating enormous data volumes that demand scalable storage infrastructure and faster network capabilities. Moreover, legacy information technology (IT) systems must be completely overhauled to support digital slide management, enable seamless integration with LISs and AI algorithms, and ensure interoperability between different viewers and platforms, a process that involves substantial technical complexity and cost.

Laboratory workflows require fundamental modifications, including changes to specimen handling, slide-scanning protocols, quality-control procedures, and pathologist reading patterns, all of which necessitate extensive staff training and adjustment periods (138). And the most critical issue is that appropriate reimbursement models remain underdeveloped. Current payment structures often fail to account for the costs of digital infrastructure, AI tool licensing, data storage, and IT support, creating economic uncertainty that slows adoption despite the technology’s potential to improve efficiency and diagnostic accuracy (139). These interconnected challenges, spanning capital investment, operational transformation, and financial sustainability, explain why digital pathology adoption has proceeded more slowly than in radiology, despite comparable technological potential (140, 141).

The CAP has established a Digital Pathology Committee to support digital pathology implementation and has published guidelines addressing validation, implementation, and quality assurance, but specific recommendations regarding reimbursement models for digital infrastructure, AI tool licensing, data storage, and IT support costs remain underdeveloped in the published literature (142, 143). However, these guidelines acknowledge that appropriate reimbursement and cost-offsetting models are necessary for proper integration of AI-based systems into anatomic pathology practice, alongside fully digital imaging platforms and IT infrastructure overhauls. Reimbursement models remain undefined for AI-assisted pathology (136). Without clear payment structures, institutions may lack financial incentive to adopt validated tools, while premature adoption of unvalidated tools to gain a competitive advantage could compromise patient safety.

### 5.12 Regulatory, legal, and ethical concerns

Current regulatory frameworks are not tailored to AI’s unique characteristics, including continuous learning, opacity of decision-making, and potential for post-deployment performance drift (136). The lack of clear liability frameworks raises questions: when an AI-assisted diagnosis proves incorrect, who bears responsibility—the pathologist, the institution, the algorithm developer, or the regulatory body that approved it (130)?

Transparency, accountability, and governance represent foundational ethical principles that are often inadequately addressed (130). Many commercial AI tools function as proprietary “black boxes” where neither the training data, algorithm architecture, nor decision-making logic is accessible to pathologists or regulators (128, 130). This opacity prevents meaningful oversight and makes it impossible to audit for bias or errors.

Data privacy concerns especially arise when training algorithms require large datasets, potentially containing identifiable patient information (132). Federated learning approaches can help by training models across institutions without centralizing data, but implementation remains technically challenging (132). Moreover, equity and fairness issues emerge when training datasets lack diversity across race, ethnicity, age, and socioeconomic status (130). For example, an algorithm trained predominantly on tissue from one demographic group may perform poorly on others, potentially exacerbating healthcare disparities (127, 129, 130).

### 5.13 Pathologist-specific concerns

The changing role of pathologists raises professional identity questions (132, 144). While AI may handle routine tasks, pathologists must develop new competencies in algorithm oversight, error recognition, and integration of AI outputs with clinical context (144). This requires training programs to evolve, yet curricula for AI literacy in pathology remain underdeveloped. Crowdsourcing and unsupervised learning trends are eliminating direct pathologist involvement in algorithm development (136). While this may accelerate progress, it risks creating tools that don’t align with clinical needs or that pathologists don’t trust or understand (136).

This affirms that the complexity gap between AI capabilities and clinical reality remains substantial (144). Current algorithms address relatively simple diagnostic problems (e.g., detecting PNI presence/absence) but fall short of the diagnostic complexity that pathologists face in routine practice, where PNI assessment integrates with tumor grade, stage, margin status, and patient-specific factors to guide management (144). These limitations emphasize that AI tools should – at least for the time being – function as decision-support systems requiring pathologist oversight rather than as autonomous diagnostic platforms. Addressing these challenges requires full-bodied external validation, standardized benchmarking, development of explainable AI approaches, pathologist education in AI literacy, and regulatory frameworks that balance innovation with patient safety (128, 130, 136).

## 6 Future vision: PNI detection and reporting in the AI-augmented pathology laboratory

**Figure 1:**
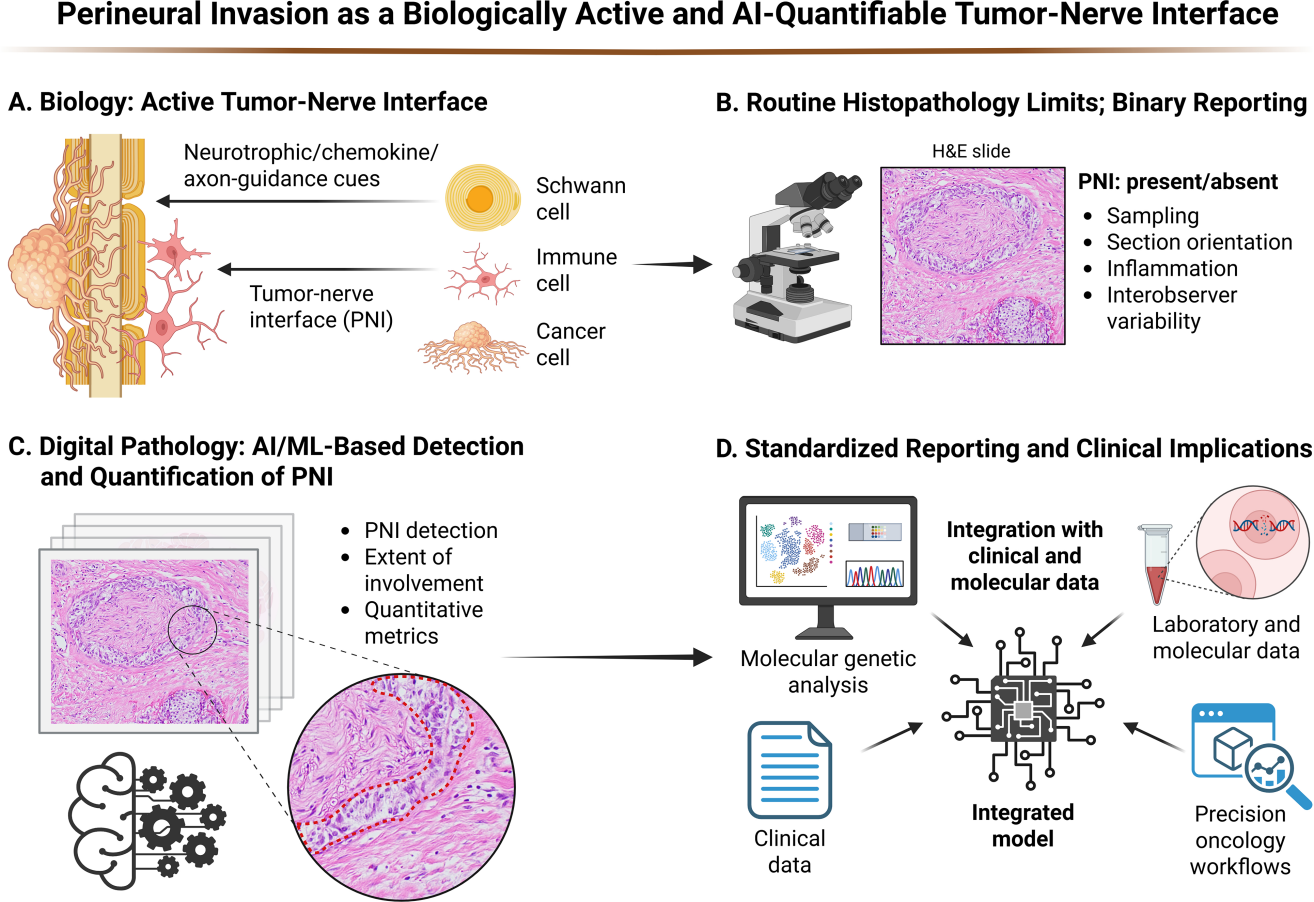
Perineural invasion as a biologically active and AI-quantifiable tumor-nerve interface. Created in BioRender (Bahmad, H. (2026) https://BioRender.com/uwjaejo).

The convergence of digital pathology, ML, and AI is set to transform PNI from an inconsistently reported histologic descriptor into a quantifiable, actionable biomarker that guides therapeutic decisions and identifies patients for novel interventions targeting the tumor-nerve axis (99, 145, 146) (**Figure 1**).

### 6.1 Near-term workflow: AI-assisted screening and quantitative assessment in routine pathology

Within the next 3–5 years, AI-assisted PNI detection will function as a first-pass screening layer integrated seamlessly into routine diagnostic workflows (145, 147). WSIs will be automatically analyzed by foundation models trained on millions of histopathology images, flagging regions of interest for pathologist review while quantifying PNI extent, spatial distribution, and severity (128, 148). This hybrid human-AI workflow addresses the fundamental tension between pathologist workload and diagnostic precision, where AI handles the labor-intensive task of screening large tissue areas at multiple section levels, while pathologists apply clinical judgment to confirm findings and integrate them with other prognostic factors (137).

The standardization enabled by AI will address long-standing variability in PNI reporting (149). Rather than binary presence/absence, structured pathology reports will include quantitative metrics, including but not limited to the number of involved nerves, maximum nerve caliber, tumor-nerve proximity measurements, and spatial localization (intratumoral versus advancing edge versus extratumoral) (8, 63). These metrics will be automatically extracted and populated into synoptic reports, creating a common language for PNI assessment across institutions and enabling meta-analyses that were previously impossible due to reporting heterogeneity (150).

### 6.2 Foundation models: a paradigm shift beyond task-specific algorithms

The transition from task-specific convolutional neural networks to foundation models represents a fundamental architectural evolution (128, 148, 151). Foundation models like UNI, CONCH (CONtrastive learning from Captions for Histopathology), GigaPath (formerly Prov-GigaPath), and BEPH (BEiT-based model Pre-training on Histopathological image) are trained on massive, diverse datasets using self-supervised learning, enabling them to develop generalizable representations of tissue architecture without requiring extensive manual annotations for each new task (128, 152–155). These models demonstrate few-shot and zero-shot learning capabilities, meaning they can adapt to new diagnostic challenges with minimal additional training data—a critical advantage for rare histologic features or emerging biomarkers (148).

For PNI specifically, foundation models will enable cross-cancer generalization, where a model trained on pancreatic cancer could be rapidly adapted to detect PNI in HNSCC, PCa, or CRC with minimal fine-tuning (107, 152). Visual-language models like CONCH integrate histopathological images with biomedical text, allowing pathologists to query slides using natural language (“show me areas of PNI with associated desmoplastic reaction”) and receive annotated results (148, 152). This conversational interface mirrors how pathologists assess multimodal information and could dramatically reduce the cognitive burden of complex case review (128, 151).

### 6.3 Multimodal integration: linking morphology to molecular mechanisms

The most transformative applications will emerge from multimodal AI that integrates histopathology with radiomics, genomics, transcriptomics, and clinical data (145, 148, 156, 157). For PNI, this means correlating morphologic features visible on H&E slides with underlying molecular drivers, such as neurotrophin signaling pathway activation, Schwann cell reprogramming signatures, immune microenvironment composition, and therapeutic vulnerabilities (158–160).

Multimodal models will enable non-invasive prediction of PNI status from preoperative imaging, guiding surgical planning and neoadjuvant therapy selection (99, 100, 157). Integration with genomic data will further reveal whether PNI-positive tumors harbor specific mutations (e.g., *FBXW7* loss-of-function in cervical cancer) that confer therapeutic vulnerabilities to targeted agents like *BET* inhibitors (161). The spatial transcriptomics revolution will map gene expression patterns directly onto tissue architecture, revealing how PNI-associated molecular programs vary across the tumor-nerve interface (150, 162). AI models trained on these multimodal datasets will identify histomorphologic phenotypes that predict molecular subtypes invisible to human observers, analogous to how deep learning predicts microsatellite instability or PD-L1 status from H&E slides (146, 163, 164).

### 6.4 PNI as a companion diagnostic for targeted therapies

As therapies targeting tumor-nerve interactions advance from preclinical models to clinical trials, AI-quantified PNI may eventually function as a companion diagnostic analogous to PD-L1 for immunotherapy or HER2 for trastuzumab (99). The presence and extent of PNI, combined with molecular signatures of active tumor-nerve crosstalk (neurotrophin receptor expression, Schwann cell activation markers), will stratify patients for enrollment in trials testing:

- *Anti-NGF monoclonal antibody Tanezumab*: Preclinical studies demonstrate that systemic NGF depletion using anti-NGF antibodies inhibits disease progression in genetically engineered PDAC models (165). Tanezumab is currently being evaluated in a phase 3 study for patients with painful bone metastases and has shown efficacy in alleviating nerve invasion and relieving nociceptive conduction in animal models (99, 166).
- *Neurotrophin pathway inhibitors (TrkA/B/C or Pan-Trk inhibitors)*: Blocking TrkA activity with small molecule inhibitors reduces proliferation, migration, and neural invasion potential of pancreatic cancer cells. GW441756 has been shown to inhibit neurite formation in response to pancreatic cancer cell-conditioned media and reduced cancer cell migration toward the dorsal root ganglia (167). Several other TrkA/B/C antagonists are also currently in early clinical trials and could be tested for pancreatic cancer-induced pain (167), such as Larotrectinib for *NTRK* fusion-positive tumors, or pan-Trk inhibitors for PNI-driven cancers (99, 168).
- *Selective RET inhibitor Selpercatinib*: The NCCN PDAC Guidelines list selpercatinib as an “other recommended regimen” for patients with *RET* gene fusion-positive PDAC (169). Interestingly, preclinical studies have demonstrated that *RET* inhibition with shRNA or small-molecule inhibitors reduced PNI in mouse models and that blocking perineurial macrophage-derived GDNF is essential for preventing neural spread of pancreatic cancer.
- *RET/VEGFR/EGFR inhibitor Vandetanib*: This is the only available RET inhibitor that selectively targets *RET*, *VEGFR*, and *EGFR* signaling. A phase 2 trial (ViP trial) evaluated vandetanib plus gemcitabine versus placebo plus gemcitabine in locally advanced or metastatic PDAC. Preclinical data showed that the combination significantly reduced tumor weight and metastases compared to either treatment alone (170). Neural invasion through GDNF secretion is a prominent feature of pancreatic cancer, with cancer cells migrating along GDNF gradients.
- *Adenosine pathway blockade*: A2A receptor antagonists may be used to reverse immunosuppression in the perineural niche (21, 44).
- *Schwann cell reprogramming inhibitors*: targeting the ITGA5-fibronectin-NGF axis (171).
- Combination strategies pairing neural modulation with immune checkpoint inhibitors to “heat” the cold TME characteristic of PNI-positive cancers (21, 44).

Additionally, AI-driven pharmacodynamic monitoring will track treatment response by quantifying changes in nerve density, tumor-nerve proximity, and immune infiltration on serial biopsies or surgical specimens (1, 20). This creates a feedback loop where therapeutic efficacy informs biomarker refinement, accelerating the identification of predictive signatures (99).

It should be noted, however, that the therapeutic targets discussed above are predominantly supported by preclinical and early clinical evidence from PDAC models. Whether these tumor-nerve signaling pathways (particularly NGF/TrkA and GDNF/RET axes) are implicated in other PNI-prone malignancies such as head and neck, colorectal, or biliary tract cancers requires further research. That is, the degree to which AI-derived PNI biomarkers can serve as predictive companion diagnostics will likely need cancer type-specific validation before broad clinical implementation.

### 6.5 Agentic AI and pathology copilots: the next frontier

Emerging agentic AI systems like PathChat and SmartPath represent a qualitative leap beyond passive decision support (128, 149). These conversational AI “copilots” can autonomously plan diagnostic workflows, retrieve relevant literature, generate differential diagnoses, and draft structured reports, all while maintaining explainability through attention maps and feature attribution (128, 149, 151). For PNI assessment, a pathology copilot might automatically detect and quantify PNI across all slides in a case, retrieve published survival data for similar PNI patterns in that cancer type, cross-reference with the patient’s genomic profile to identify targetable pathways, generate a structured report integrating morphologic, molecular, and prognostic information, and even suggest clinical trial eligibility based on PNI characteristics. This augmented intelligence approach preserves pathologist oversight while dramatically expanding the scope and depth of diagnostic assessment (137, 145). Therefore, the pathologist’s role evolves from manual feature detection to high-level synthesis, quality assurance, and clinical contextualization—tasks that require human judgment and cannot be automated (104, 144).

### 6.6 Addressing implementation challenges: the path to clinical adoption

Realizing this vision requires overcoming substantial technical, regulatory, and cultural barriers (141, 145, 149). Key priorities include broad external validation across diverse patient populations, institutions, and scanning platforms to ensure models generalize beyond their training environments (128, 141). Current foundation models show poor zero-shot performance on new datasets, highlighting the need for federated learning approaches that enable collaborative model training without centralizing patient data (152, 172). There is a dire need for explainable AI development that uses attention mechanisms, saliency maps, and SHAP values to make model predictions interpretable and trustworthy for pathologists (12, 99, 152, 163). However, “Black-box” algorithms that cannot explain their reasoning will face resistance from clinicians and regulators (102, 141, 172). The FDA’s evolving approach to software as a medical device provides a foundation, but PNI-specific guidance will still be needed (147, 149). Another issue that needs to be addressed is the pathologists’ education in AI literacy, including understanding algorithmic limitations, recognizing failure modes, and integrating AI outputs into the clinical context (104, 145, 173). Training residency and fellowship programs must evolve to prepare the next generation of pathologists for AI-augmented practice (151, 174). Lastly, ensuring equitable access is essential so that AI tools benefit all patients, not only those cared for at well-resourced academic centers (102, 149). In this context, open-source foundation models and federated learning frameworks offer a promising path to democratize access while preserving patient privacy (152, 172).

## 7 Conclusions

Over the coming years, PNI assessment has the potential to evolve substantially from current practice. Emerging AI-based foundation models may eventually enable automated, comprehensive neural mapping of cancer specimens, quantifying nerve density, tumor-nerve interactions, and immune contexture at increasingly granular resolution. As these morphologic capabilities mature, they could be progressively integrated with spatial transcriptomics, genomics, proteomics, and metabolomics data, moving toward the long-term goal of constructing digital representations of individual tumors. While the concept of *in silico* testing of therapeutic strategies before clinical implementation remains aspirational, continued advances in computational pathology and multi-omics integration are laying the groundwork for such approaches. However, significant technical, regulatory, and validation difficulties must be overcome before these technologies can be widely adopted in routine clinical practice.

For patients with PNI-positive cancers, treatment decisions will be guided by AI-generated risk scores that integrate morphologic PNI extent, molecular pathway activation, immune microenvironment composition, and predicted response to nerve-targeted therapies. Clinical trials will use AI-quantified PNI as a stratification variable and pharmacodynamic endpoint, accelerating the development of effective interventions.

Realizing this transformative vision will require overcoming substantial barriers, including infrastructure limitations, regulatory gaps, liability uncertainties, workforce adaptation, and validation challenges, but the convergence of foundation models, multimodal data integration, and AI-driven analytics positions computational pathology to fundamentally reshape cancer diagnosis and personalized treatment within the coming decade.

## References

[1] Shaikh M Shirodkar S Doshi G . Unraveling the role of perineural invasion in cancer progression across multiple tumor types. Med Oncol. (2025) 42:283. doi: 10.1007/s12032-025-02855-6. PMID: 40569482

[2] Liebig C Ayala G Wilks JA Berger DH Albo D . Perineural invasion in cancer: a review of the literature. Cancer. (2009) 115:3379–91. doi: 10.1002/cncr.24396. PMID: 19484787

[3] Egevad L Delahunt B Samaratunga H Tsuzuki T Olsson H Ström P . Interobserver reproducibility of perineural invasion of prostatic adenocarcinoma in needle biopsies. Virchows Arch. (2021) 478:1109–16. doi: 10.1007/s00428-021-03039-z. PMID: 33534005 PMC8203540

[4] Dunn M Morgan MB Beer TW . Perineural invasion: identification, significance, and a standardized definition. Dermatol Surg. (2009) 35:214–21. doi: 10.1111/j.1524-4725.2008.34412.x. PMID: 19215258

[5] van Wyk HC Going J Horgan P McMillan DC . The role of perineural invasion in predicting survival in patients with primary operable colorectal cancer: a systematic review. Crit Rev Oncol Hematol. (2017) 112:11–20. doi: 10.1016/j.critrevonc.2017.02.005. PMID: 28325252

[6] Wang M Pu N Bo X Chen F Zhou Y Cheng Q . Significance and mechanisms of perineural invasion in Malignant tumors. Front Oncol. (2025) 15:1572396. doi: 10.3389/fonc.2025.1572396. PMID: 40421086 PMC12104087

[7] Schouten TJ Kroon VJ Besselink MG Bosscha K Busch OR Crobach A . Perineural invasion is an important prognostic factor in patients with radically resected (R0) and node-negative (pN0) pancreatic cancer. Ann Surg. (2025) 282:1083–91. doi: 10.1097/sla.0000000000006320. PMID: 38708885

[8] Crippa S Pergolini I Javed AA Honselmann KC Weiss MJ Di Salvo F . Implications of perineural invasion on disease recurrence and survival after pancreatectomy for pancreatic head ductal adenocarcinoma. Ann Surg. (2022) 276:378–85. doi: 10.1097/sla.0000000000004464. PMID: 33086324

[9] Yuan H Zhang Y Liu F Wu Y Huang X Liu X . Exploring the biological mechanism and clinical value of perineural invasion in pancreatic cancer. Cancer Lett. (2025) 613:217515. doi: 10.1016/j.canlet.2025.217515. PMID: 39892698

[10] Tao ZY Chu G Su YX . The prognostic role of perineural invasion for survival in head and neck squamous cell carcinoma: a systematic review and meta-analysis. Cancers (Basel). (2024) 16. doi: 10.3390/cancers16142514. PMID: 39061154 PMC11274576

[11] Zhu J Zhou R Wang Y Yu M . Perineural invasion as a prognostic factor in head and neck squamous cell carcinoma: a systematic review and meta-analysis. Acta Otolaryngol. (2019) 139:1038–43. doi: 10.1080/00016489.2019.1655167. PMID: 31464544

[12] Bakst RL Glastonbury CM Parvathaneni U Katabi N Hu KS Yom SS . Perineural invasion and perineural tumor spread in head and neck cancer. Int J Radiat Oncol Biol Phys. (2019) 103:1109–24. doi: 10.1016/j.ijrobp.2018.12.009. PMID: 30562546

[13] Vogel JD Felder SI Bhama AR Hawkins AT Langenfeld SJ Shaffer VO . The american society of colon and rectal surgeons clinical practice guidelines for the management of colon cancer. Dis Colon Rectum. (2022) 65:148–77. doi: 10.1097/dcr.0000000000002323. PMID: 34775402

[14] Benson AB Venook AP Adam M Chang G Chen Y-J Ciombor KK . Colon cancer, version 3.2024, NCCN Clinical Practice Guidelines in Oncology. J Natl Compr Cancer Netw. (2024) 22:e240029. doi: 10.6004/jnccn.2024.0029. PMID: 38862008

[15] Benson AB Venook AP Adam M Chang G Chen Y-J Ciombor KK . NCCN Guidelines® Insights: Rectal cancer, version 3.2024: featured updates to the NCCN Guidelines. J Natl Compr Cancer Netw. (2024) 22:366–75. doi: 10.6004/jnccn.2024.0041. PMID: 39151454

[16] Ström P Nordström T Delahunt B Samaratunga H Grönberg H Egevad L . Prognostic value of perineural invasion in prostate needle biopsies: a population-based study of patients treated by radical prostatectomy. J Clin Pathol. (2020) 73:630–5. doi: 10.1136/jclinpath-2019-206300. PMID: 32034057 PMC7513266

[17] Siech C Wenzel M Grosshans N Cano Garcia C Humke C Koll FJ . The association between lymphovascular or perineural invasion in radical prostatectomy specimen and biochemical recurrence. Cancers (Basel). (2024) 16. doi: 10.3390/cancers16213648. PMID: 39518086 PMC11545596

[18] Teramoto Y Wang Y Miyamoto H . Risk stratification by quantification of perineural cancer invasion on prostate needle core biopsy: should it be counted? J Urol. (2023) 210:639–48. doi: 10.1097/ju.0000000000003618. PMID: 37433144

[19] Harnden P Shelley MD Clements H Coles B Tyndale-Biscoe RS Naylor B . The prognostic significance of perineural invasion in prostatic cancer biopsies: a systematic review. Cancer. (2007) 109:13–24. doi: 10.1002/cncr.22388. PMID: 17123267

[20] Ebrahim NAA Soliman SMA Othman MO Tahoun NS . Molecular mechanisms and clinical significance of perineural invasion in Malignancies: the pivotal role of tumor-associated Schwann cells in cancer progression and metastasis. Med Oncol. (2025) 42:171. doi: 10.1007/s12032-025-02729-x. PMID: 40259163

[21] Li S Xiao H . Perineural invasion as a neuro-immune niche in head and neck cancer: mechanisms of immune evasion and therapeutic implications. Front Immunol. (2025) 16:1719675. doi: 10.3389/fimmu.2025.1719675. PMID: 41445745 PMC12722934

[22] Bakst RL Xiong H Chen CH Deborde S Lyubchik A Zhou Y . Inflammatory monocytes promote perineural invasion via CCL2-mediated recruitment and cathepsin B expression. Cancer Res. (2017) 77:6400–14. doi: 10.1158/0008-5472.Can-17-1612. PMID: 28951461 PMC5831809

[23] Massey PR Wang DM Murad F Mulvaney P Moore K Okhovat JP . Extensive perineural invasion vs nerve caliber to assess cutaneous squamous cell carcinoma prognosis. JAMA Dermatol. (2023) 159:1332–8. doi: 10.1001/jamadermatol.2023.3703. PMID: 37851425 PMC10585586

[24] Bahmad HF Wegner C Nuraj J Avellan R Gonzalez J Mendez T . Perineural invasion in breast cancer: a comprehensive review. Cancers (Basel). (2025) 17. doi: 10.3390/cancers17121900. PMID: 40563551 PMC12190579

[25] Bahmad HF Gogola S Rejzer M Stoyanov K Gomez AS Valencia AK . Unraveling the mysteries of perineural invasion in benign and Malignant conditions. Curr Oncol. (2023) 30:8948–72. doi: 10.3390/curroncol30100647. PMID: 37887547 PMC10605475

[26] Zhou ZH Xu GF Zhang WJ Zhao HB Wu YY . Reevaluating significance of perineural invasion in gastric cancer based on double immunohistochemical staining. Arch Pathol Lab Med. (2014) 138:229–34. doi: 10.5858/arpa.2012-0669-OA. PMID: 24476520

[27] Mohamed S Callanan D Sheahan P Feeley L . Significance of location and extent of perineural invasion in early-stage oral cavity squamous cell carcinoma. Histopathology. (2025) 86:993–1000. doi: 10.1111/his.15406. PMID: 39762203 PMC11964579

[28] Chen H Wang C Chen Z Huang T Lin Y Chen J . The depth of perineural invasion is an independent prognostic factor for stage II colorectal cancer. BMC Cancer. (2024) 24:433. doi: 10.1186/s12885-024-12206-9. PMID: 38589842 PMC11003015

[29] Cho SS Park JW Kang GH Kim JH Bae JM Han SW . Prognostic impact of extramural lymphatic, vascular, and perineural invasion in stage II colon cancer: a comparison with intramural invasion. Dis Colon Rectum. (2023) 66:366–73. doi: 10.1097/dcr.0000000000002339. PMID: 35333785

[30] Burgart L Chopp W Jain D . Protocol for the examination of Excisional Biopsy Specimens From Patients With Primary Carcinoma of the Colon and Rectum. College of American Pathologists (2021).

[31] Burgart L Chopp W Jain D . Protocol for the Examination of Specimens From Patients With Carcinoma of the Gallbladder. College of American Pathologists (2021).

[32] Burgart L Chopp W Jain D . Protocol for the Examination of Specimens From Patients With Carcinoma of the Pancreas. College of American Pathologists (2021).

[33] Burgart L Chopp W Jain D . Protocol for the Examination of Specimens From Patients With Carcinoma of the Stomach. College of American Pathologists (2023).

[34] Paner G Srigley J Pettus J Giannico G Sirintrapun J Harik L . Protocol for the Examination of Prostate Needle Biopsies From Patients With Carcinoma of the Prostate Gland: Specimen Level Reporting. College of American Pathologists (2021).

[35] Seethala R Shon W Balzer B Duvvuri U Gharavi N Lydiatt W . Protocol for the Examination of Specimens from Patients with Cutaneous Squamous Cell Carcinoma of the Head and Neck. College of American Pathologists (2022).

[36] Grignon DJ . Prostate cancer reporting and staging: needle biopsy and radical prostatectomy specimens. Mod Pathol. (2018) 31:S96–109. doi: 10.1038/modpathol.2017.167. PMID: 29297497

[37] Egevad L Judge M Delahunt B Humphrey PA Kristiansen G Oxley J . Dataset for the reporting of prostate carcinoma in core needle biopsy and transurethral resection and enucleation specimens: recommendations from the International Collaboration on Cancer Reporting (ICCR). Pathology. (2019) 51:11–20. doi: 10.1016/j.pathol.2018.10.003. PMID: 30477882

[38] Hruban R Adsay N Esposito I Klöppel G Zamboni G Furukawa T . Pancreatic ductal adenocarcinoma. In: Digestive System Tumours. WHO Classification of Tumours online: International Agency for Research on Cancer (2019). Available online at: https://tumourclassification.iarc.who.int/chaptercontent/31/126/169.

[39] Kench J Kristiansen G Berney D Netto G Marzo A Litjens G . Prostatic acinar adenocarcinoma. In: Urinary and Male Genital Tumours, 5th ed. WHO Classification of Tumours online: International Agency for Research on Cancer (2022). Available online at: https://tumourclassification.iarc.who.int/chaptercontent/36/100/553.

[40] Messina J Calonje J Zalaudek I Fernandez-Figueras M . Squamous cell carcinomas. In: Skin Tumours, 5th ed. WHO Classification of Tumours online: International Agency for Research on Cancer (2023). Available online at: https://tumourclassification.iarc.who.int/chaptercontent/64/35/778.

[41] Nadal A Bishop J Brandwein-Weber M Stenman G Zidar N . Conventional squamous cell carcinoma. In: Head and Neck Tumours, 5th ed. WHO Classification of Tumours online: International Agency for Research on Cancer (2023). Available online at: https://tumourclassification.iarc.who.int/chaptercontent/52/45.

[42] Nagtegaal I Arends M Salto-Tellez M . Colorectal adenocarcinoma. In: Digestive System Tumours. WHO Classification of Tumours online: International Agency for Research on Cancer (2019). Available online at: https://tumourclassification.iarc.who.int/chaptercontent/31/62/169.

[43] Robinson TP Kaiser K Lark M Ruedinger B Robb BW Morgan T . NCCN guideline concordance in colon and rectal cancer patients within a comprehensive health system. Am J Surg. (2025) 240:116114. doi: 10.1016/j.amjsurg.2024.116114. PMID: 39671967 PMC11745911

[44] Xu J Yao H Wang J Jin Y Chang W Li L . Perineural invasion and the “cold” tumor microenvironment in pancreatic cancer: mechanisms of crosstalk and therapeutic opportunities. Front Immunol. (2025) 16:1650117. doi: 10.3389/fimmu.2025.1650117. PMID: 40909268 PMC12404959

[45] Melgarejo da Rosa M Clara Sampaio M Virgínia Cavalcanti Santos R Sharjeel M Araújo C Galdino da Rocha Pitta M . Unveiling the pathogenesis of perineural invasion from the perspective of neuroactive molecules. Biochem Pharmacol. (2021) 188:114547. doi: 10.1016/j.bcp.2021.114547. PMID: 33838132

[46] Yang Z Li H Wang J Gao W Zhao Q Meng Q . CCL2/CCR2 axis promotes perineural invasion of salivary adenoid cystic carcinoma via ITGβ5-mediated nerve-tumor interaction. Biochim Biophys Acta Mol Basis Dis. (2025) 1871:167484. doi: 10.1016/j.bbadis.2024.167484. PMID: 39222826

[47] He S He S Chen CH Deborde S Bakst RL Chernichenko N . The chemokine (CCL2-CCR2) signaling axis mediates perineural invasion. Mol Cancer Res. (2015) 13:380–90. doi: 10.1158/1541-7786.Mcr-14-0303. PMID: 25312961 PMC4336839

[48] Zhang Y Yuan Y Wang Y Ye Z Liu T Lv G . The bridging role of Schwann cells in the interaction between tumors and the nervous system: a potential target for cancer therapy. Mol Cancer Res. (2025) 23:494–502. doi: 10.1158/1541-7786.Mcr-25-0124. PMID: 40202770

[49] Azam SH Pecot CV . Cancer’s got nerve: Schwann cells drive perineural invasion. J Clin Invest. (2016) 126:1242–4. doi: 10.1172/jci86801. PMID: 26999601 PMC4811122

[50] He Y Chen Z Yang L Qiao S Su Z Ding F . The supporting role of Schwann cells in perineural invasion of pancreatic ductal adenocarcinoma. Front Pharmacol. (2025) 16:1540027. doi: 10.3389/fphar.2025.1540027. PMID: 40567365 PMC12187733

[51] Zhang W He R Yang W Zhang Y Yuan Q Wang J . Autophagic Schwann cells promote perineural invasion mediated by the NGF/ATG7 paracrine pathway in pancreatic cancer. J Exp Clin Cancer Res. (2022) 41:48. doi: 10.1186/s13046-021-02198-w. PMID: 35109895 PMC8809009

[52] Chen G Zheng Z Sun H You J Chu J Gao J . Dedifferentiated Schwann cells promote perineural invasion mediated by the PACAP paracrine signalling in cervical cancer. J Cell Mol Med. (2023) 27:3692–705. doi: 10.1111/jcmm.17897. PMID: 37830980 PMC10718160

[53] Gao X Wang Q Huang T Xu C Yang X Zhang L . Cervical cancer-produced neuromedin-B reprograms Schwann cells to initiate perineural invasion. Cell Death Dis. (2024) 15:636. doi: 10.1038/s41419-024-07030-9. PMID: 39214988 PMC11364772

[54] Chen MM Gao Q Ning H Chen K Gao Y Yu M . Integrated single-cell and spatial transcriptomics uncover distinct cellular subtypes involved in neural invasion in pancreatic cancer. Cancer Cell. (2025) 43:1656–1676.e10. doi: 10.1016/j.ccell.2025.06.020. PMID: 40680743

[55] Cao J Zhang C Chen T Tian R Sun S Yu X . Plexin-B1 and semaphorin 4D cooperate to promote cutaneous squamous cell carcinoma cell proliferation, migration and invasion. J Dermatol Sci. (2015) 79:127–36. doi: 10.1016/j.jdermsci.2015.05.002. PMID: 26051877

[56] Jurcak NR Rucki AA Muth S Thompson E Sharma R Ding D . Axon guidance molecules promote perineural invasion and metastasis of orthotopic pancreatic tumors in mice. Gastroenterology. (2019) 157:838–850.e6. doi: 10.1053/j.gastro.2019.05.065. PMID: 31163177 PMC6707836

[57] Kato S Kubota K Shimamura T Shinohara Y Kobayashi N Watanabe S . Semaphorin 4D, a lymphocyte semaphorin, enhances tumor cell motility through binding its receptor, plexinB1, in pancreatic cancer. Cancer Sci. (2011) 102:2029–37. doi: 10.1111/j.1349-7006.2011.02053.x. PMID: 21812859

[58] Tassone P Caruso C White M Tavares Dos Santos H Galloway T Dooley L . The role of matrixmetalloproteinase-2 expression by fibroblasts in perineural invasion by oral cavity squamous cell carcinoma. Oral Oncol. (2022) 132:106002. doi: 10.1016/j.oraloncology.2022.106002. PMID: 35779484

[59] Huang C Li Y Guo Y Zhang Z Lian G Chen Y . MMP1/PAR1/SP/NK1R paracrine loop modulates early perineural invasion of pancreatic cancer cells. Theranostics. (2018) 8:3074–86. doi: 10.7150/thno.24281. PMID: 29896303 PMC5996366

[60] Na’ara S Amit M Gil Z . L1CAM induces perineural invasion of pancreas cancer cells by upregulation of metalloproteinase expression. Oncogene. (2019) 38:596–608. doi: 10.1038/s41388-018-0458-y. PMID: 30171263

[61] Krauss T Gürcinar IH Bourquain U Hieber M Krohmer EN Wu N . Pancreatic cancer cells infiltrate nerves through TGFbeta1-driven perineural epithelial-to-mesenchymal-like transdifferentiation. Neoplasia. (2025) 60:101126. doi: 10.1016/j.neo.2025.101126. PMID: 39842382 PMC11763858

[62] Ji F Chen H Li H Zhang J Li S Wang P . A subcellular spatial atlas illuminates the microenvironmental remodeling of perineural invasion in distal cholangiocarcinoma. J Hematol Oncol. (2026) 19:6. doi: 10.1186/s13045-025-01773-4. PMID: 41508044 PMC12784525

[63] Gasparini G Crippa S Lena MS Belfiori G Aleotti F Di Salvo F . Prognostic validation of perineural invasion severity score in pancreatic cancer: A prospective study. Ann Surg. (2025). doi: 10.1097/sla.0000000000006920. PMID: 40856533

[64] Schorn S Fritz A Kaissis G Gaida MM Steiger K Jäger C . Neural invasion severity is a strong predictor of local recurrence in pancreatic ductal adenocarcinoma. Surgery. (2025) 180:109018. doi: 10.1016/j.surg.2024.109018. PMID: 39798180

[65] Tzorakoleftheraki SE Kotoula V Karakatsoulis G Markou K Pervana S Vlachtsis K . Deciphering site-specific histopathological parameters with potential clinical value in head and neck squamous cell carcinomas. Oncol Lett. (2025) 30:569. doi: 10.3892/ol.2025.15315. PMID: 41103535 PMC12522186

[66] Liebig C Ayala G Wilks J Verstovsek G Liu H Agarwal N . Perineural invasion is an independent predictor of outcome in colorectal cancer. J Clin Oncol. (2009) 27:5131–7. doi: 10.1200/jco.2009.22.4949. PMID: 19738119 PMC2773472

[67] Fujita S Shimoda T Yoshimura K Yamamoto S Akasu T Moriya Y . Prospective evaluation of prognostic factors in patients with colorectal cancer undergoing curative resection. J Surg Oncol. (2003) 84:127–31. doi: 10.1002/jso.10308. PMID: 14598355

[68] Quah HM Chou JF Gonen M Shia J Schrag D Landmann RG . Identification of patients with high-risk stage II colon cancer for adjuvant therapy. Dis Colon Rectum. (2008) 51:503–7. doi: 10.1007/s10350-008-9246-z. PMID: 18322753

[69] Bell PD Teramoto Y Gurung PMS Numbere N Yang Z Miyamoto H . The clinical significance of perineural invasion by prostate cancer on needle core biopsy: Involvement of single versus multiple sextant sites. Arch Pathol Lab Med. (2022) 146:1252–7. doi: 10.5858/arpa.2021-0248-OA. PMID: 35020791

[70] Lanipekun OK Wang Y Miyamoto H . Clinical significance of the relative location of perineural cancer invasion on prostate biopsy: Detection within 1-mm of the core tip as an independent prognosticator. Hum Pathol. (2025) 168:106015. doi: 10.1016/j.humpath.2025.106015. PMID: 41389896

[71] Brown MD Hart CA Sachdeva A Faulkner C Wedge D Clarke NW . (2021). Meeting abstract: 2021 GenitourinaryCancers Symposium. J Clin Oncol. 39:253. doi: 10.1200/JCO.2021.39.6_suppl.253

[72] Gadducci A Pistolesi S Cosio S Naccarato AG . Is perineural invasion a novel prognostic factor useful to tailor adjuvant treatment in patients treated with primary surgery for cervical and vulvar carcinoma? Anticancer Res. (2020) 40:3031–7. doi: 10.21873/anticanres.14283. PMID: 32487596

[73] Chen X Duan H Zhao H He F Yin L Liu Y . Perineural invasion in cervical cancer: A multicenter retrospective study. Eur J Surg Oncol. (2024) 50:108313. doi: 10.1016/j.ejso.2024.108313. PMID: 38579659

[74] Cai G Zhang S Gao S Deng T Huang H Feng Y . What is the impact of perineural invasion on the prognosis of cervical cancer: a systematic review and meta-analysis. BMC Cancer. (2025) 25:491. doi: 10.1186/s12885-025-13838-1. PMID: 40098102 PMC11917148

[75] Memarzadeh S Natarajan S Dandade DP Ostrzega N Saber PA Busuttil A . Lymphovascular and perineural invasion in the parametria: a prognostic factor for early-stage cervical cancer. Obstet Gynecol. (2003) 102:612–9. doi: 10.1016/s0029-7844(03)00569-6. PMID: 12962952

[76] Wan T Tu H Liu L Huang H Feng Y Liu J . Perineural invasion should be regarded as an intermediate-risk factor for recurrence in surgically treated cervical cancer: A propensity score matching study. Dis Markers. (2021) 2021:1375123. doi: 10.1155/2021/1375123. PMID: 34394773 PMC8357507

[77] Li X Yang X Lin S Cong H Liu Y Wang Y . Perineural invasion in cervical cancer. Cancer Lett. (2025) 616:217561. doi: 10.1016/j.canlet.2025.217561. PMID: 39956383

[78] Santoro A Angelico G Travaglino A Inzani F Arciuolo D Valente M . Prognostic role of perineural invasion in vulvar squamous cell carcinoma: A systematic review and meta-analysis. Eur J Surg Oncol. (2022) 48:2354–9. doi: 10.1016/j.ejso.2022.06.031. PMID: 35811178

[79] Salcedo MP Sood AK Dos Reis R Ramalingam P Chen C Frumovitz M . Perineural invasion (PNI) in vulvar carcinoma: A review of 421 cases. Gynecol Oncol. (2019) 152:101–5. doi: 10.1016/j.ygyno.2018.10.035. PMID: 30396690

[80] Zheng Z Li X Chen G Chen J Zhu X Teng Y . Transcriptome analyses reveal new insights on key determinants of perineural invasion in high-grade serous ovarian cancer. Front Cell Dev Biol. (2023) 11:1109710. doi: 10.3389/fcell.2023.1109710. PMID: 37799274 PMC10548129

[81] Crosbie EJ Kitson SJ McAlpine JN Mukhopadhyay A Powell ME Singh N . Endometrial cancer. Lancet. (2022) 399:1412–28. doi: 10.1016/s0140-6736(22)00323-3. PMID: 35397864

[82] Ni T Huang T Gu SL Wang J Liu Y Sun X . DRG neurons promote perineural invasion of endometrial cancer via GluR2. J Cancer. (2020) 11:2518–28. doi: 10.7150/jca.40055. PMID: 32201522 PMC7066017

[83] Shen FZ Zhang BY Feng YJ Jia ZX An B Liu CC . Current research in perineural invasion of cholangiocarcinoma. J Exp Clin Cancer Res. (2010) 29:24. doi: 10.1186/1756-9966-29-24. PMID: 20219134 PMC2851676

[84] Zhang Z Zhou Y Hu K Wang D Wang Z Huang Y . Perineural invasion as a prognostic factor for intrahepatic cholangiocarcinoma after curative resection and a potential indication for postoperative chemotherapy: a retrospective cohort study. BMC Cancer. (2020) 20:270. doi: 10.1186/s12885-020-06781-w. PMID: 32228636 PMC7106692

[85] Tan X Sivakumar S Bednarsch J Wiltberger G Kather JN Niehues J . Nerve fibers in the tumor microenvironment in neurotropic cancer-pancreatic cancer and cholangiocarcinoma. Oncogene. (2021) 40:899–908. doi: 10.1038/s41388-020-01578-4. PMID: 33288884 PMC7862068

[86] Kami S Kojima M Kudo M Kamiya H Une N Sasahara Y . Clinicopathological significance and prognostic impact of perineural invasion in intrahepatic cholangiocarcinoma: a single-center, retrospective study. Hum Pathol. (2025) 166:105952. doi: 10.1016/j.humpath.2025.105952. PMID: 41077256

[87] Wei T Zhang XF He J Popescu I Marques HP Aldrighetti L . Prognostic impact of perineural invasion in intrahepatic cholangiocarcinoma: multicentre study. Br J Surg. (2022) 109:610–6. doi: 10.1093/bjs/znac098. PMID: 35511599

[88] Kawashima J Akabane M Tsilimigras DI Woldesenbet S Khalil M Endo Y . Oncologic impact of perineural invasion in perihilar cholangiocarcinoma: an international multicenter study. HPB (Oxford). (2025) 27:1158–67. doi: 10.1016/j.hpb.2025.05.010. PMID: 40494701

[89] Lv TR Hu HJ Liu F Ma WJ Jin YW Li FY . The role of extra-hepatic bile duct resection in patients with gallbladder carcinoma with peri-neural invasion: A ten-year experience in China. Eur J Surg Oncol. (2023) 49:1009–15. doi: 10.1016/j.ejso.2022.12.018. PMID: 36604233

[90] Zhao J Zhu J Yang Z Zhai Y Zhao C Lu Z . Extracellular vesicle-mediated O-GlcNAcase transfer drives neuronal necroptosis to facilitate gallbladder cancer perineural invasion. Cancer Res. (2026) 86:1392–413. doi: 10.1158/0008-5472.Can-25-2237. PMID: 41460724 PMC13012253

[91] Deng J You Q Gao Y Yu Q Zhao P Zheng Y . Prognostic value of perineural invasion in gastric cancer: a systematic review and meta-analysis. PloS One. (2014) 9:e88907. doi: 10.1371/journal.pone.0088907. PMID: 24586437 PMC3931634

[92] Zhao B Lv W Mei D Luo R Bao S Huang B . Perineural invasion as a predictive factor for survival outcome in gastric cancer patients: a systematic review and meta-analysis. J Clin Pathol. (2020) 73:544–51. doi: 10.1136/jclinpath-2019-206372. PMID: 31980559

[93] Chang K Song B Do IG Koo DH Lee HW Son BH . Venous invasion and perineural invasion as upstaging and poor prognostic factors in N0 gastric cancers. Anticancer Res. (2021) 41:5803–10. doi: 10.21873/anticanres.15397. PMID: 34732454

[94] Woodham BL Chmelo J Donohoe CL Madhavan A Phillips AW . Prognostic significance of lymphatic, venous and perineural invasion after neoadjuvant chemotherapy in patients with gastric adenocarcinoma. Ann Surg Oncol. (2020) 27:3296–304. doi: 10.1245/s10434-020-08389-7. PMID: 32219726 PMC7410853

[95] Blumenthaler AN Newhook TE Ikoma N Estrella JS Blum Murphy M Das P . Concurrent lymphovascular and perineural invasion after preoperative therapy for gastric adenocarcinoma is associated with decreased survival. J Surg Oncol. (2021) 123:911–22. doi: 10.1002/jso.26367. PMID: 33400838 PMC7906958

[96] Kurtz KA Hoffman HT Zimmerman MB Robinson RA . Perineural and vascular invasion in oral cavity squamous carcinoma: increased incidence on re-review of slides and by using immunohistochemical enhancement. Arch Pathol Lab Med. (2005) 129:354–9. doi: 10.5858/2005-129-354-paviio. PMID: 15737030

[97] Guo JA Hoffman HI Shroff SG Chen P Hwang PG Kim DY . Pan-cancer transcriptomic predictors of perineural invasion improve occult histopathologic detection. Clin Cancer Res. (2021) 27:2807–15. doi: 10.1158/1078-0432.Ccr-20-4382. PMID: 33632928 PMC8127360

[98] Kartasalo K Ström P Ruusuvuori P Samaratunga H Delahunt B Tsuzuki T . Detection of perineural invasion in prostate needle biopsies with deep neural networks. Virchows Arch. (2022) 481:73–82. doi: 10.1007/s00428-022-03326-3. PMID: 35449363 PMC9226086

[99] Shi DD Guo JA Hoffman HI Su J Mino-Kenudson M Barth JL . Therapeutic avenues for cancer neuroscience: translational frontiers and clinical opportunities. Lancet Oncol. (2022) 23:e62–74. doi: 10.1016/s1470-2045(21)00596-9. PMID: 35114133 PMC9516432

[100] Borsekofsky S Tsuriel S Hagege RR Hershkovitz D . Perineural invasion detection in pancreatic ductal adenocarcinoma using artificial intelligence. Sci Rep. (2023) 13:13628. doi: 10.1038/s41598-023-40833-y. PMID: 37604973 PMC10442355

[101] Lee LY Yang CH Lin YC Hsieh YH Chen YA Chang MD . A domain knowledge enhanced yield based deep learning classifier identifies perineural invasion in oral cavity squamous cell carcinoma. Front Oncol. (2022) 12:951560. doi: 10.3389/fonc.2022.951560. PMID: 36353548 PMC9638412

[102] Brouwer NPM Lord AC Terlizzo M Bateman AC West NP Goldin R . Interobserver variation in the classification of tumor deposits in rectal cancer-is the use of histopathological characteristics the way to go? Virchows Arch. (2021) 479:1111–8. doi: 10.1007/s00428-021-03197-0. PMID: 34480612 PMC8724135

[103] Littleford SE Baird A Rotimi O Verbeke CS Scott N . Interobserver variation in the reporting of local peritoneal involvement and extramural venous invasion in colonic cancer. Histopathology. (2009) 55:407–13. doi: 10.1111/j.1365-2559.2009.03397.x. PMID: 19817891

[104] Dang C Qi Z Xu T Gu M Chen J Wu J . Deep learning-powered whole slide image analysis in cancer pathology. Lab Invest. (2025) 105:104186. doi: 10.1016/j.labinv.2025.104186. PMID: 40306572

[105] Wang S Yang DM Rong R Zhan X Xiao G . Pathology image analysis using segmentation deep learning algorithms. Am J Pathol. (2019) 189:1686–98. doi: 10.1016/j.ajpath.2019.05.007. PMID: 31199919 PMC6723214

[106] Khened M Kori A Rajkumar H Krishnamurthi G Srinivasan B . A generalized deep learning framework for whole-slide image segmentation and analysis. Sci Rep. (2021) 11:11579. doi: 10.1038/s41598-021-90444-8. PMID: 34078928 PMC8172839

[107] Li X Huang J Wang C Yu X Zhao T Huang C . Expectation-maximization algorithm leads to domain adaptation for a perineural invasion and nerve extraction task in whole slide digital pathology images. Med Biol Eng Comput. (2023) 61:457–73. doi: 10.1007/s11517-022-02711-z. PMID: 36496513

[108] Ahmedt-Aristizabal D Armin MA Denman S Fookes C Petersson L . A survey on graph-based deep learning for computational histopathology. Comput Med Imaging Graph. (2022) 95:102027. doi: 10.1016/j.compmedimag.2021.102027. PMID: 34959100

[109] Pu T Sun J Yue J Zhang Z Li H Ren G . Machine learning-based pathomics signature for perineural invasion in colorectal cancer. Med Sci Monit. (2025) 31:e951110. doi: 10.12659/msm.951110. PMID: 41069091 PMC12519896

[110] Bera K Schalper KA Rimm DL Velcheti V Madabhushi A . Artificial intelligence in digital pathology - new tools for diagnosis and precision oncology. Nat Rev Clin Oncol. (2019) 16:703–15. doi: 10.1038/s41571-019-0252-y. PMID: 31399699 PMC6880861

[111] Xie T Huang A Yan H Ju X Xiang L Yuan J . Artificial intelligence: illuminating the depths of the tumor microenvironment. J Transl Med. (2024) 22:799. doi: 10.1186/s12967-024-05609-6. PMID: 39210368 PMC11360846

[112] Zhong J Huang T Jiang R Zhou Q Wu G Zeng Y . MRI-based habitat, intra-, and peritumoral machine learning model for perineural invasion prediction in rectal cancer. Abdom Radiol (NY). (2025) 51 (2):545–57. doi: 10.1007/s00261-025-05095-4. PMID: 40603736

[113] Zhang YY Mao HM Wei CG Chen T Zhao WL Chen LY . Development and validation of a biparametric MRI deep learning radiomics model with clinical characteristics for predicting perineural invasion in patients with prostate cancer. Acad Radiol. (2024) 31:5054–65. doi: 10.1016/j.acra.2024.07.013. PMID: 39043515

[114] Sun Y Li Y Li M Hu T Wang J . Radiomics analysis using machine learning to predict perineural invasion in pancreatic cancer. BMC Cancer. (2025) 25:1480. doi: 10.1186/s12885-025-14806-5. PMID: 41034781 PMC12487371

[115] Yu J Chen C Lu M Fang X Li J Zhu M . Computed tomography-based fully automated artificial intelligence model to predict extrapancreatic perineural invasion in pancreatic ductal adenocarcinoma. Int J Surg. (2024) 110:7656–70. doi: 10.1097/js9.0000000000001604. PMID: 39806736 PMC11634086

[116] Weusthof C Burkart S Semmelmayer K Stögbauer F Feng B Khorani K . Establishment of a machine learning model for the risk assessment of perineural invasion in head and neck squamous cell carcinoma. Int J Mol Sci. (2023) 24. doi: 10.3390/ijms24108938. PMID: 37240283 PMC10218829

[117] Gao X Cui J Wang L Wang Q Ma T Yang J . The value of machine learning based radiomics model in preoperative detection of perineural invasion in gastric cancer: a two-center study. Front Oncol. (2023) 13:1205163. doi: 10.3389/fonc.2023.1205163. PMID: 37388227 PMC10303108

[118] Hanna MG Olson NH Zarella M Dash RC Herrmann MD Furtado LV . Recommendations for performance evaluation of machine learning in pathology: a concept paper from the College of American Pathologists. Arch Pathol Lab Med. (2024) 148:e335–61. doi: 10.5858/arpa.2023-0042-CP. PMID: 38041522

[119] Collins GS Moons KGM Dhiman P Riley RD Beam AL Van Calster B . TRIPOD+AI statement: updated guidance for reporting clinical prediction models that use regression or machine learning methods. Bmj. (2024) 385:e078378. doi: 10.1136/bmj-2023-078378. PMID: 38626948 PMC11019967

[120] Deng S Huang D Han X Zhang H Wang H Mao G . A novel MRI-based habitat analysis and deep learning for predicting perineural invasion in prostate cancer: a two-center study. BMC Cancer. (2025) 25:1367. doi: 10.1186/s12885-025-14759-9. PMID: 40849663 PMC12374478

[121] He J Wang X Zhu P Wang X Zhang Y Zhao J . Identification and validation of an explainable early-stage chronic kidney disease prediction model: a multicenter retrospective study. EClinicalMedicine. (2025) 84:103286. doi: 10.1016/j.eclinm.2025.103286. PMID: 40567347 PMC12192353

[122] Xue B Li D Lu C King CR Wildes T Avidan MS . Use of machine learning to develop and evaluate models using preoperative and intraoperative data to identify risks of postoperative complications. JAMA Netw Open. (2021) 4:e212240. doi: 10.1001/jamanetworkopen.2021.2240. PMID: 33783520 PMC8010590

[123] Pan Y Wei M Jin M Liang Y Yi T Tu J . An interpretable machine learning model based on optimal feature selection for identifying CT abnormalities in patients with mild traumatic brain injury. EClinicalMedicine. (2025) 82:103192. doi: 10.1016/j.eclinm.2025.103192. PMID: 40242564 PMC12002887

[124] Tan G Wang WQ Yuan T Liu JJ Xie ZH Zhang ZY . Machine learning prediction of perineural invasion in intrahepatic cholangiocarcinoma. Eur J Surg Oncol. (2025) 51:110203. doi: 10.1016/j.ejso.2025.110203. PMID: 40449386

[125] Liu Z Luo C Chen X Feng Y Feng J Zhang R . Noninvasive prediction of perineural invasion in intrahepatic cholangiocarcinoma by clinicoradiological features and computed tomography radiomics based on interpretable machine learning: a multicenter cohort study. Int J Surg. (2024) 110:1039–51. doi: 10.1097/js9.0000000000000881. PMID: 37924497 PMC10871628

[126] Qi Z Yuan H Li Q Chen P Li D Chen K . An MRI-based fusion model for preoperative prediction of perineural invasion status in patients with intrahepatic cholangiocarcinoma. World J Surg Oncol. (2025) 23:164. doi: 10.1186/s12957-025-03819-w. PMID: 40287750 PMC12032683

[127] Evans H Snead D . Why do errors arise in artificial intelligence diagnostic tools in histopathology and how can we minimize them? Histopathology. (2024) 84:279–87. doi: 10.1111/his.15071. PMID: 37921030

[128] Cheng CH Wong CC . The role of artificial intelligence-based foundation models and “copilots” in cancer pathology: potential and challenges. J Exp Clin Cancer Res. (2025) 45:2. doi: 10.1186/s13046-025-03592-4. PMID: 41316486 PMC12763834

[129] Evans H Snead D . Understanding the errors made by artificial intelligence algorithms in histopathology in terms of patient impact. NPJ Digit Med. (2024) 7:89. doi: 10.1038/s41746-024-01093-w. PMID: 38600151 PMC11006652

[130] Chauhan C Gullapalli RR . Ethics of AI in pathology: current paradigms and emerging issues. Am J Pathol. (2021) 191:1673–83. doi: 10.1016/j.ajpath.2021.06.011. PMID: 34252382 PMC8485059

[131] Kwon D . How artificial intelligence is transforming pathology. Nature. (2025) 641:1342–4. doi: 10.1038/d41586-025-01576-0. PMID: 40410341

[132] Nakagawa K Moukheiber L Celi LA Patel M Mahmood F Gondim D . AI in pathology: what could possibly go wrong? Semin Diagn Pathol. (2023) 40:100–8. doi: 10.1053/j.semdp.2023.02.006. PMID: 36882343 PMC13175311

[133] Keith J . The burnout in Canadian pathology initiative. Arch Pathol Lab Med. (2022) 147:568–76. doi: 10.5858/arpa.2021-0200-OA. PMID: 35939795

[134] Smith SM Liauw D Dupee D Barbieri AL Olson K Parkash V . Burnout and disengagement in pathology: a prepandemic survey of pathologists and laboratory professionals. Arch Pathol Lab Med. (2023) 147:808–16. doi: 10.5858/arpa.2022-0073-OA. PMID: 36191345

[135] Bellahsen-Harrar Y Lubrano M Lépine C Beaufrère A Bocciarelli C Brunet A . Exploring the risks of over-reliance on AI in diagnostic pathology. What lessons can be learned to support the training of young pathologists? PloS One. (2025) 20:e0323270. doi: 10.1371/journal.pone.0323270. PMID: 40875775 PMC12393786

[136] Cheng JY Abel JT Balis UGJ McClintock DS Pantanowitz L . Challenges in the development, deployment, and regulation of artificial intelligence in anatomic pathology. Am J Pathol. (2021) 191:1684–92. doi: 10.1016/j.ajpath.2020.10.018. PMID: 33245914

[137] Niazi MKK Parwani AV Gurcan MN . Digital pathology and artificial intelligence. Lancet Oncol. (2019) 20:e253–61. doi: 10.1016/s1470-2045(19)30154-8. PMID: 31044723 PMC8711251

[138] Schwen LO Kiehl TR Carvalho R Zerbe N Homeyer A . Digitization of pathology labs: a review of lessons learned. Lab Invest. (2023) 103:100244. doi: 10.1016/j.labinv.2023.100244. PMID: 37657651

[139] Aggarwal A Bharadwaj S Corredor G Pathak T Badve S Madabhushi A . Artificial intelligence in digital pathology - time for a reality check. Nat Rev Clin Oncol. (2025) 22:283–91. doi: 10.1038/s41571-025-00991-6. PMID: 39934323

[140] Reis-Filho JS Kather JN . Overcoming the challenges to implementation of artificial intelligence in pathology. J Natl Cancer Inst. (2023) 115:608–12. doi: 10.1093/jnci/djad048. PMID: 36929936 PMC10248832

[141] Verghese G Lennerz JK Ruta D Ng W Thavaraj S Siziopikou KP . Computational pathology in cancer diagnosis, prognosis, and prediction - present day and prospects. J Pathol. (2023) 260:551–63. doi: 10.1002/path.6163. PMID: 37580849 PMC10785705

[142] Hanna MG Pantanowitz L Evans AJ . Overview of contemporary guidelines in digital pathology: what is available in 2015 and what still needs to be addressed? J Clin Pathol. (2015) 68:499–505. doi: 10.1136/jclinpath-2015-202914. PMID: 25979986

[143] Evans AJ Salama ME Henricks WH Pantanowitz L . Implementation of whole slide imaging for clinical purposes: issues to consider from the perspective of early adopters. Arch Pathol Lab Med. (2017) 141:944–59. doi: 10.5858/arpa.2016-0074-OA. PMID: 28440660

[144] Stenzinger A Alber M Allgäuer M Jurmeister P Bockmayr M Budczies J . Artificial intelligence and pathology: from principles to practice and future applications in histomorphology and molecular profiling. Semin Cancer Biol. (2022) 84:129–43. doi: 10.1016/j.semcancer.2021.02.011. PMID: 33631297

[145] Marra A Morganti S Pareja F Campanella G Bibeau F Fuchs T . Artificial intelligence entering the pathology arena in oncology: current applications and future perspectives. Ann Oncol. (2025) 36:712–25. doi: 10.1016/j.annonc.2025.03.006. PMID: 40307127

[146] Takamatsu M . Transforming histologic assessment: artificial intelligence in cancer diagnosis and personalized treatment. Br J Cancer. (2025) 133:1765–75. doi: 10.1038/s41416-025-03206-y. PMID: 40993310 PMC12689690

[147] Geaney A O’Reilly P Maxwell P James JA McArt D Salto-Tellez M . Translation of tissue-based artificial intelligence into clinical practice: from discovery to adoption. Oncogene. (2023) 42:3545–55. doi: 10.1038/s41388-023-02857-6. PMID: 37875656 PMC10673711

[148] Jang HJ Lee SH . AI-driven digital pathology: deep learning and multimodal integration for precision oncology. Int J Mol Sci. (2025) 27. doi: 10.3390/ijms27010379. PMID: 41516254 PMC12785522

[149] Shaktah LA Carrero ZI Hewitt KJ Gustav M Cecchini M Foersch S . Application of artificial intelligence and digital tools in cancer pathology. Lancet Digit Health. (2025) 7:100933. doi: 10.1016/j.landig.2025.100933. PMID: 41241581

[150] Hijazi A Bifulco C Baldin P Galon J . Digital pathology for better clinical practice. Cancers (Basel). (2024) 16. doi: 10.3390/cancers16091686. PMID: 38730638 PMC11083211

[151] Waqas A Bui MM Glassy EF El Naqa I Borkowski P Borkowski AA . Revolutionizing digital pathology with the power of generative artificial intelligence and foundation models. Lab Invest. (2023) 103:100255. doi: 10.1016/j.labinv.2023.100255. PMID: 37757969

[152] Hacking S . Foundation models in pathology: bridging AI innovation and clinical practice. J Clin Pathol. (2025) 78:433–5. doi: 10.1136/jcp-2024-209910. PMID: 40355256

[153] Yang Z Wei T Liang Y Yuan X Gao R Xia Y . A foundation model for generalizable cancer diagnosis and survival prediction from histopathological images. Nat Commun. (2025) 16:2366. doi: 10.1038/s41467-025-57587-y. PMID: 40064883 PMC11894166

[154] Skourti E . Foundation models in clinical oncology. Nat Cancer. (2024) 5:1790. doi: 10.1038/s43018-024-00837-7. PMID: 39690213

[155] Xu H Wang M Shi D Qin H Zhang Y Liu Z . When multiple instance learning meets foundation models: advancing histological whole slide image analysis. Med Image Anal. (2025) 101:103456. doi: 10.1016/j.media.2025.103456. PMID: 39842326

[156] Boehm KM Khosravi P Vanguri R Gao J Shah SP . Harnessing multimodal data integration to advance precision oncology. Nat Rev Cancer. (2022) 22:114–26. doi: 10.1038/s41568-021-00408-3. PMID: 34663944 PMC8810682

[157] Shao J Ma J Zhang Q Li W Wang C . Predicting gene mutation status via artificial intelligence technologies based on multimodal integration (MMI) to advance precision oncology. Semin Cancer Biol. (2023) 91:1–15. doi: 10.1016/j.semcancer.2023.02.006. PMID: 36801447

[158] Lipkova J Chen RJ Chen B Lu MY Barbieri M Shao D . Artificial intelligence for multimodal data integration in oncology. Cancer Cell. (2022) 40:1095–110. doi: 10.1016/j.ccell.2022.09.012. PMID: 36220072 PMC10655164

[159] Yang H Yang M Chen J Yao G Zou Q Jia L . Multimodal deep learning approaches for precision oncology: a comprehensive review. Brief Bioinform. (2024) 26. doi: 10.1093/bib/bbae699. PMID: 39757116 PMC11700660

[160] He X Liu X Zuo F Shi H Jing J . Artificial intelligence-based multi-omics analysis fuels cancer precision medicine. Semin Cancer Biol. (2023) 88:187–200. doi: 10.1016/j.semcancer.2022.12.009. PMID: 36596352

[161] Wan T Deng T Peng X Cai G Huang H Ling Y . Comprehensive multi-omic characterization of perineural invasion in cervical cancer reveals diagnostic markers, molecular drivers, and therapeutic strategies. Cancer Res. (2025) 86 (6):1514–25. doi: 10.1158/0008-5472.Can-25-0149. PMID: 41379570

[162] Zhang M Yin L . Explainable artificial intelligence for multi-modal cancer analysis: from genomics to immunology. Crit Rev Oncol Hematol. (2025) 219:105040. doi: 10.1016/j.critrevonc.2025.105040. PMID: 41290079

[163] Khosravi P Fuchs TJ Ho DJ . Artificial intelligence-driven cancer diagnostics: enhancing radiology and pathology through reproducibility, explainability, and multimodality. Cancer Res. (2025) 85:2356–67. doi: 10.1158/0008-5472.Can-24-3630. PMID: 40598940

[164] Acs B Rantalainen M Hartman J . Artificial intelligence as the next step towards precision pathology. J Intern Med. (2020) 288:62–81. doi: 10.1111/joim.13030. PMID: 32128929

[165] Saloman JL Singhi AD Hartman DJ Normolle DP Albers KM Davis BM . Systemic depletion of nerve growth factor inhibits disease progression in a genetically engineered model of pancreatic ductal adenocarcinoma. Pancreas. (2018) 47:856–63. doi: 10.1097/mpa.0000000000001090. PMID: 29975347 PMC6044729

[166] Peng T Guo Y Gan Z Ling Y Xiong J Liang X . Nerve growth factor (NGF) encourages the neuroinvasive potential of pancreatic cancer cells by activating the Warburg effect and promoting tumor derived exosomal miRNA-21 expression. Oxid Med Cell Longev. (2022) 2022:8445093. doi: 10.1155/2022/8445093. PMID: 36285300 PMC9588358

[167] Bapat AA Munoz RM Von Hoff DD Han H . Blocking nerve growth factor signaling reduces the neural invasion potential of pancreatic cancer cells. PloS One. (2016) 11:e0165586. doi: 10.1371/journal.pone.0165586. PMID: 27792755 PMC5085053

[168] Wang Y Ye Z Yuan Y Wang C Chen G Zhang Y . Sensory neuro-tumor crosstalk: therapeutic opportunities and emerging frontiers in cancer neuroscience. Biochim Biophys Acta Rev Cancer. (2025) 1880:189464. doi: 10.1016/j.bbcan.2025.189464. PMID: 41016671

[169] Citterio C Vecchia S Mordenti P Anselmi E Ratti M Guasconi M . Targeting advanced pancreatic ductal adenocarcinoma: a practical overview. Gastroenterol Insights. (2025) 16:26. doi: 10.3390/gastroent16030026. PMID: 41725453

[170] Middleton G Palmer DH Greenhalf W Ghaneh P Jackson R Cox T . Vandetanib plus gemcitabine versus placebo plus gemcitabine in locally advanced or metastatic pancreatic carcinoma (ViP): a prospective, randomised, double-blind, multicentre phase 2 trial. Lancet Oncol. (2017) 18:486–99. doi: 10.1016/s1470-2045(17)30084-0. PMID: 28259610

[171] Liu Y Han G Feng K Lin X Zhong W Liu Y . ITGA5-expressing tumor cells interact with Schwann cells to drive nerve growth factor-mediated immunosuppression of NK cells. Mol Ther. (2025) 33:5591–610. doi: 10.1016/j.ymthe.2025.07.043. PMID: 40734271 PMC12628158

[172] Fei Du R Lloret Carbonell E Huang J Liu S Wang X Shen D . Ethics of foundation models in computational pathology: overview of contemporary issues and future implications. IEEE Trans Med Imaging. (2025) 44:4098–115. doi: 10.1109/tmi.2025.3551913. PMID: 40095828

[173] Rakha EA Toss M Shiino S Gamble P Jaroensri R Mermel CH . Current and future applications of artificial intelligence in pathology: a clinical perspective. J Clin Pathol. (2021) 74:409–14. doi: 10.1136/jclinpath-2020-206908. PMID: 32763920

[174] Sagiv C Hadar O Najjar A Pahnke J . Artificial intelligence in surgical pathology - where do we stand, where do we go? Eur J Surg Oncol. (2025) 51:109541. doi: 10.1016/j.ejso.2024.109541. PMID: 39694737

